# Potential alternative and novel biomarkers for paediatric MAFLD: exploratory evidence from a Chinese cohort

**DOI:** 10.1186/s12876-025-03619-2

**Published:** 2025-01-22

**Authors:** Fan Yang, Mengyuan Hu, Lulian Xu, Xiaowei Zheng, Lihong Zhu, Le Zhang, Haoyang Zhang

**Affiliations:** 1 Department of Paediatric Laboratory, Affiliated Children’s Hospital of Jiangnan University, Wuxi Children’s Hospital, Wuxi, P. R. China; 2 Wuxi School of Medicine, Jiangnan University, Wuxi, P. R. China; 3 Department of Paediatrics, Jinhua Maternal and Child Health Hospital, Jinhua, P. R. China; 4 Department of Experimental Medical Science, Lund University, Lund, Sweden

**Keywords:** MAFLD, Paediatrics, Liver steatosis, Biomarker

## Abstract

**Background:**

While the associations between pediatric non-obese metabolic dysfunction-associated fatty liver disease (MAFLD) and multiple diagnostic biomarkers are well-established, the role of a broader range of blood-based, urine-based, and body composition-based biomarkers for monitoring MAFLD are needed.

**Methods:**

A pediatric cohort was established in Wuxi, China. We measured body composition biomarkers, blood-based and urine-based biomarkers, and liver stiffness in participants to diagnose MAFLD and identify alternative and novel potential biomarkers for MAFLD. Body mass index (BMI), high-density lipoprotein cholesterol (HDLC), triglycerides, glucose, systolic blood pressure (SBP), diastolic blood pressure (DBP), and waist perimeter were used as MAFLD diagnostic biomarkers. To identify alternative biomarkers, we performed correlation analysis to determine biomarkers exhibited strong correlation (|*r*| > 0.8, *p-value* < 0.05) with diagnostic biomarkers. To identify novel potential biomarkers, we performed regression analysis to determine biomarkers associated with MAFLD (*p-value* < 0.05 in stepwise multivariate regression) among the remaining biomarkers that are not related to the diagnostic biomarkers.

**Results:**

Out of 1,108 participants who completed all examinations (*N* biomarker = 91), 113 participants were diagnosed with MAFLD (prevalence: 14.99% in boys and 5.18% in girls). 27 biomarkers that were strongly correlated with diagnostic biomarkers were identified as alternative biomarkers. A multivariate logistic regression analysis identified 9 novel potential biomarkers including 5 blood-based biomarkers (plateletocrit, calcium, insulin, AST/ALT ratio, total bilirubin), urine pH, and body fat measurements in the arm, leg, and thigh.

**Conclusions:**

This study illustrated the characteristics and potential alternative and novel biomarkers of MAFLD based on a Chinese paediatric cohort. These findings posed new paths in guiding the prevention and early diagnosis and prevention.

**Trial registration:**

This trial was registered in the Chinese Clinical Trials Registry (ChiCTR2400080508). The date of first registration, 01/31/2024. Retrospectively registered.

**Supplementary Information:**

The online version contains supplementary material available at 10.1186/s12876-025-03619-2.

## Introduction

Currently, metabolic-associated fatty liver disease (MAFLD) affects more than one-third of the world’s population. Approximately 3–10% of the general paediatric population suffers from MAFLD, with this percentage significantly increasing to 80% among overweight and obese children [1]. In sharp contrast to the high prevalence rate, public awareness of paediatric MAFLD is notably insufficient. There are mechanisms that could explain the underlying disease in these patients, but most of them are concealed. In the early stages, these patients lack distinct clinical symptoms, making MAFLD easy to overlook. Another significant factor affecting MAFLD awareness is the low detection rate in routine screenings. While liver biopsy remains the gold standard for MAFLD diagnosis, its invasive nature and cost constraints restrict its clinical application [2, 3]. Other imaging methods, such as ultrasound imaging (B-mode ultrasonography) and magnetic resonance elastography, are also limited for routine screening because of their subjectivity, high costs, and lower detection rates [4, 5].

However, over time, MAFLD can not only progress to hepatocellular carcinoma but also result in extrahepatic complications such as hypothyroidism, osteoporosis, and cognitive dysfunction [6, 7]. Currently, the primary treatment approach for MAFLD involves lifestyle interventions, such as increased physical activity and dietary adjustments. Therefore, having a clear understanding of the factors influencing paediatric MAFLD and undertaking early interventions is of paramount importance. There are evidences that high-fat diets, sedentarism and excessive intake of carbohydrates contribute to metabolic syndrome and type 2 diabetes mellitus (T2DM) in children and adolescents [8–10]. Additionally, factors such as obesity, hepatic fat accumulation induced by insulin resistance; and prenatal, environmental, and genetic factors, potentially contribute to the development of paediatric MAFLD [11–13].

Previous research has focused mainly on the relationships between single diseases or specific biomarkers (e.g., genetic, microbiome, and metabolomic biomarkers) and MAFLD. However, relatively few studies have investigated diverse biomarkers, such as body compositions and blood / urine– based biomarkers, which could provide comprehensive and useful information for a more holistic assessment of MAFLD. For example, crucial elements in the diagnosis of MAFLD include blood biomarkers such as high-density lipoprotein cholesterol (HDLC), alanine aminotransferase (ALT), lipid concentrations, and glucose (GLU), along with changes in body composition such as an increase in the waist-to-hip ratio (WHR) and a decrease in muscle mass [14, 15]. Moreover, urinary biomarkers such as protein, uric acid, and bilirubin not only reflect liver inflammation, damage, or metabolic abnormalities but are also closely linked to the development of MAFLD [16]. Therefore, a meticulous analysis of these component biomarkers enables early and accurate detection and diagnosis of MAFLD, facilitating prompt intervention and treatment for patients and aiding in reducing the risk of disease progression.

Furthermore, existing cohort research on paediatric MAFLD has focused mostly on specific groups, such as overweight and obese individuals [17, 18], lacking a comprehensive representation of the general paediatric population. When establishing a paediatric MAFLD cohort study, diagnosis typically relies on MRI [19, 20], and compared with the use of FibroScan devices, its diagnostic rate is relatively low. Current cohort research concerning risk factors for paediatric MAFLD primarily revolves around common blood biochemical markers such as triglycerides (TG), aspartate aminotransferase (AST), and low-density lipoprotein cholesterol (LDLC) [18, 21].

According to the international expert consensus statement [22], the screening and diagnosis of MAFLD in children involve identifying intrahepatic fat accumulation through hepatic steatosis imaging (controlled attenuation parameter [CAP]) or blood biomarkers (e.g., HDLC, TG, GLU, systolic blood pressure [SBP], diastolic blood pressure [DBP]), along with metabolic risk factors (e.g., body mass index [BMI], waist perimeter). While multiple highly relevant biomarkers for screening and diagnosing MAFLD (referred to as “diagnostic biomarkers”) have been established, further investigation is needed to explore the role of various biomarkers in the development of MAFLD. This study aimed to identify biomarkers correlated with either one diagnostic biomarker that can serve as “alternative biomarkers”. Given the complexity of MAFLD, there may be novel potential biomarkers unrelated to any single diagnostic biomarker but associated with the concept of MAFLD. Therefore, we also identified “novel potential biomarkers” associated with MAFLD that are independent of any diagnostic biomarkers. This study provides a scientific basis for optimizing the management system and intervention measures for children at high risk of MAFLD.

## Materials and methods

### Design and setting

The study was designed as a cross-sectional study based on baseline data from a cohort study set at a primary school (a campus of an educational group) in Wuxi city, which started in March 2023. The baseline data were collected from March 13th to March 27th.

A total of 1,475 students from grades 1 to 6 (aged 6 to 13) agreed to participate in the study and completed all the examinations. In this study, efforts were made to address potential sources of bias. To maintain the precision of our determinations, each instrument underwent thorough inspection and calibration. All personnel engaged in the determinations received professional training and had over two years of work experience. To ensure transparency and thoroughness in our reporting, we registered our study protocol with the Chinese Clinical Trial Registry (No. ChiCTR2400080508, Release date: 01/31/2024), encompassing all planned analyses and outcomes. This prespecification serves to deter selective reporting of results influenced by data considerations.

### Study participants

The educational group, the most populous school in Wuxi city by student enrollment, spans all districts with six campuses. In this study, one campus of the school was randomly chosen, and tests were conducted on all the students. This study initially included 1475 students aged 6 to 13 years who underwent health examinations (blood pressure measurements were only taken for students aged 8 years and above [*N* = 674]) and provided written informed consent from their parents or legal guardians. However, 367 of them were excluded for having incomplete examinations (excluding blood pressure); therefore, 1,108 children contributed to the subsequent analysis.

The subjects were divided into non-MAFLD and MAFLD groups on the basis of the presence of hepatic steatosis and fulfilment of at least one of the following conditions: overweight or obesity (including abdominal obesity), prediabetes or T2DM, and metabolic dysregulation [22].

Hepatic steatosis was identified using the criterion of a CAP value ≥ 238 dB/m [23]. For the classification of overweight and obesity, children under 5 years with a BMI greater than 2 standard deviations (SD) above the WHO growth reference median are considered overweight, and those with a BMI greater than 3 SD are considered obese. For children aged 5 to 15 years, a BMI greater than 1 SD above the median indicates overweight, and a BMI greater than 2 SD above the median indicates obesity [24]. Abdominal obesity was defined as having a waist perimeter greater than the 90th percentile [25]. According to international criteria for the diagnosis of prediabetes or T2DM, this study used a fasting blood GLU concentration ≥ 5.6 mmol/L as the basis for analysis [26]. If the results indicate that the plasma TG concentration is > 90th percentile, the plasma HDLC concentration is ≤ 10th percentile, the SBP or DBP is > 90th percentile, and the TG-to-HDLC ratio is > 2.25 (applicable to children aged 2 to 9 years); if the SBP is > 130 mmHg or DBP is > 85mmHg, the plasma TG concentration is > 150 mg/dL, the plasma HDLC concentration is < 40 mg/dL, and the TG-to-HDLC ratio is > 2.25 (applicable to children between 10 and 15 years), it indicates the presence of metabolic risk abnormalities [22, 27].

**Fig. 1:**
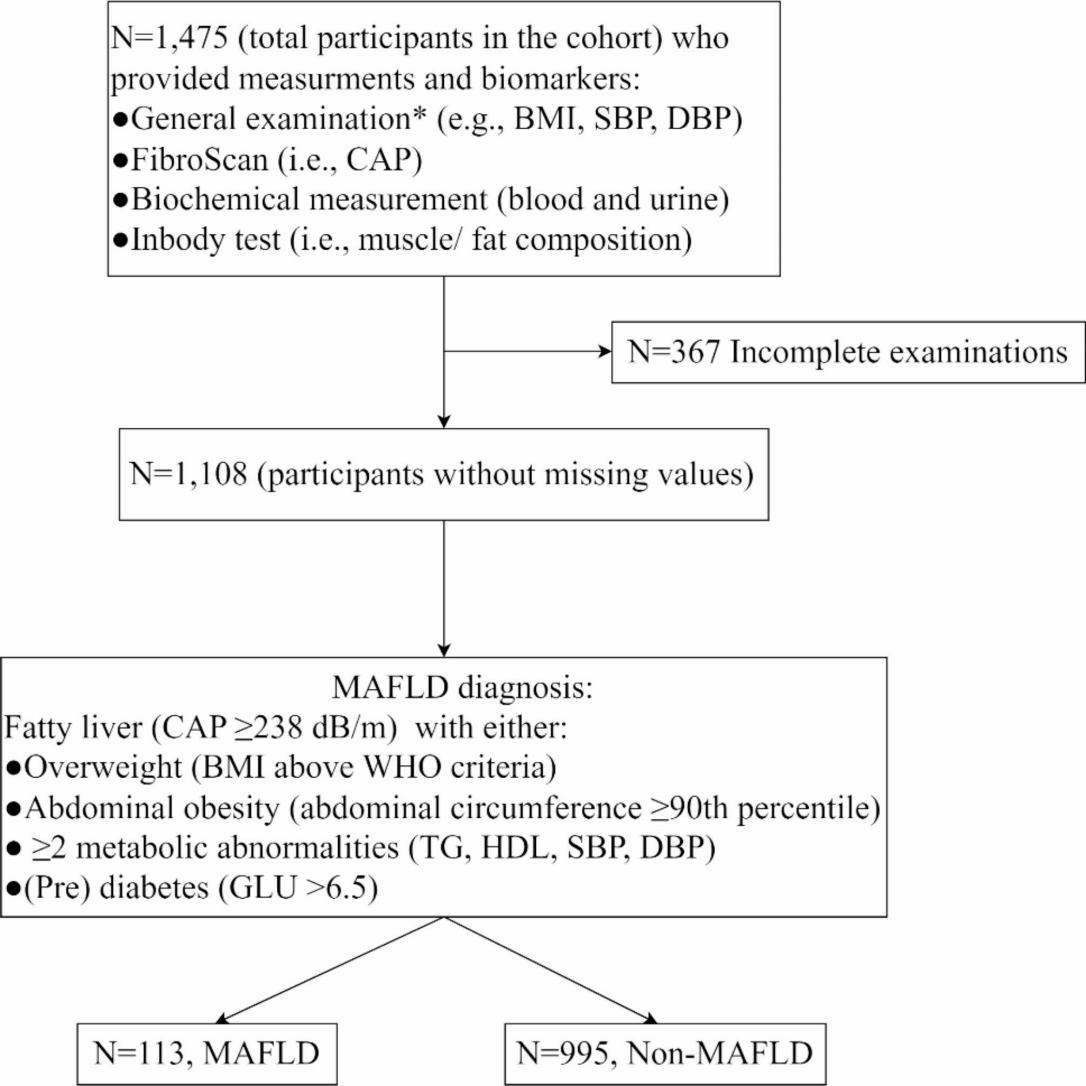
Flowchart of the study. The flowchart sample selection and MAFLD diagnosis criteria * Measurements of blood pressure were only conducted for children aged 8 and above (*N* = 674)

Finally, out of 1,108 children, 113 were diagnosed with MAFLD, and 94 had complete blood pressure data. Figure 1 shows the cohort and research question flow chart.

### Biomarker measurement

All examinations were performed at the Children’s Hospital affiliated with Jiangnan University. Before undergoing tests, the students were assigned unique ID codes. Data on height, weight, blood pressure, and biochemical tests were tracked and recorded through their respective IDs. Body composition and FibroScan examination data were downloaded immediately after the tests were completed on the same day and uploaded to the database. All the collected data were ultimately integrated and compiled using the students’ IDs for identification. The specific methods have been detailed in a previous article published by us [28].

Under standardized conditions, with participants not wearing shoes or heavy clothing, the data were collected. Measurements of height and weight were taken for each child, with height recorded in centimetres and weight recorded in kilograms (keep one decimal place). BMI (BMI = weight [kg]/height [m]^2^) was calculated from these height and weight measurements.

The blood pressure measuring device used was an OMRON HBP-1320 (HBP-1320-E) (Omron Healthcare Europe B.V. Scorpius 33, 2132 LR Hoofddorp, Netherlands). The subjects sat in chairs with a supportive back, with their arm and elbow resting on the table, legs uncrossed, and feet flat on the floor. After the samples rested for 5 min, the measurements began. Blood pressure was measured on the subjects’ right arms, ensuring that the arm was level with the heart during measurement. Blood pressure was measured three times at 3-minute intervals, and the average of the last two measurements obtained with each cuff was used for the analysis. This procedure was conducted in the presence of two trained observers who had experience measuring blood pressure in pediatric populations. Importantly, blood pressure measurements were taken only for children aged 8 years and above.

Blood was collected via venipuncture before the children had eaten, and approximately 10 to 12 ml of midstream urine from the first morning void was collected for biochemical tests. After collection, the blood was stored at 4℃. Trained professionals at the hospital separated the blood and urine samples collected on the day of the examination, with 1 ml and 300 µl units of blood and 4.5 ml units of urine, respectively. In the whole blood samples, white blood cells (WBC), GLU, platelet (PLT), AST, ALT, and 44 other biomarkers were measured. In the urine samples, parameters such as potential of hydrogen (pH), ketone concentrations, creatinine, the urinary protein-creatinine ratio, and protein were measured (the normal ranges and measurement units are shown in Table S1). After completing the tests, trained personnel collected and packaged the remaining biological samples uniformly. Blood samples were frozen at -80 °C, whereas urine samples were stored at -30 °C.

Bioelectrical impedance (In-body 370, Seoul, South Korea) was utilized to measure the body composition of paediatric individuals. This project was performed by two professionally trained and experienced radiologists. The instrument was calibrated before the measurement, and the subjects were confirmed to be in a calm state on an empty stomach. Each subject’s number and height were measured and entered into the system. The subjects were asked to remove their shoes and socks, placing their feet flat on the electrodes at the bottom of the device and holding the handle with their hands. The associated biomarkers for these components included the WHR, the percent body fat (PBF), and 29 other biomarkers (as shown in Table S1).

Transient elastography via a FibroScan HANDY^®^ (Echosens, Paris, France) equipped with an M probe (3.5 MHz) was performed to evaluate the degree of hepatic fibrosis by two trained doctors. Before the measurement, the subjects maintained an empty stomach and a calm state. During the measurement, the subjects were supine, lying flat on the test bed with their right hand behind their head and their body slightly bent to the left, fully exposing the costal space in the right lobe of the chest and liver. The doctor set the stool on the right side of the subject, facing the subject’s chest and the instrument screen, and attached the M + probe coated with conductive gel to the subject’s costal space (intercostal spaces No. 7–9 were selected) [29]. The low-frequency vibration wave emitted by the probe is measured, and at least ten valid personal measurements are eventually [30]. Both the CAP value (in dB/m) and the E-score (in kPA) were recorded for each child [5].

### Statistical analysis

Statistical analysis was performed using R 4.2.2. After descriptive statistics were calculated for the overall population on the basis of the presence of MAFLD, the data were further stratified by sex to analyse the characteristics of the participants of sex. Continuous variables are presented as the mean ± SD, whereas categorical variables are presented as numbers (*N*) and percentages (%). Intergroup differences in normally and non-normally distributed continuous variables were compared using the t-test and Mann-Whitney U test, respectively. The Fisher test was applied to compare the difference in proportions for categorical variables. A total of 1108 participants were included in the data analysis. A total of 1108 participants were included in the data analysis, excluding those with blood pressure data (674 participants).

Alternative biomarkers were determined as biomarkers exhibited strong correlations (Pearson’s|*r*| > 0.8 and *p-value* < 0.05) with diagnostic biomarkers (i.e., BMI, HDLC, TG, GLU, SBP, DBP, and waist perimeter). Given the complexity of MAFLD, we also identify “novel potential biomarkers” associated with MAFLD that are not correlated with any diagnostic biomarkers. To do so, for the remaining biomarkers (non-diagnostic and non-alternative ones), we first identified independent biomarkers (|*r*| < 0.8 or *p-value* ≥ 0.05) within four groups: body composition, blood biochemistry, blood measurements, and urine measurements. Next, we conducted univariate and stepwise multivariate logistic regression analyses sequentially, using age and sex as covariates, to identify novel potential biomarkers. The effect size was indicated by the OR (95% CI). Statistical significance was denoted at a *p-value* < 0.05. The analysis pipeline was documented at https://jinqiao-cohort.readthedocs.io/en/latest/.

## Results

### Population characteristics and prevalence of MAFLD

After applying the eligibility criteria, we described the characteristics of 1,108 participants, among whom 113 (10.20%) individuals had MAFLD, while the remaining 995 (89.80%) had non-MAFLD (look Fig. 1 for further details). As shown in Table 1 and Table S2, the mean age was 9.47 ± 1.80 years, with a roughly equal sex distribution (males: 51.17%, females: 48.83%). The average BMI Z-score was significantly greater in the MAFLD group than in the non-MAFLD group (0.69 vs. 0.30). When stratified by sex, the average BMI Z-score in the MAFLD group remained notably higher than that in the non-MAFLD group, with values of 0.65 vs. 0.30 for males and 0.79 vs. 0.29 for females, respectively (as shown in Table 1 and Table S3).

**Table 1:** Baseline characteristics of the participants

Characteristics	Total	MAFLD	Non-MAFLD	*p*-value
	(*N* = 1108)	(*N* = 113)	(*N* = 995)	
Age (years)	9.47 ± 1.80	10.36 ± 1.51	9.37 ± 1.80	< 0.001
Sex				< 0.001
Male	567(51.17%)	85(75.22%)	482(48.44%)	
Female	541(48.83%)	28(24.78%)	513(51.56%)	
Body mass index Z-score	0.34 ± 1.50	0.69 ± 1.64	0.30 ± 1.47	0.019
High-density lipoprotein cholesterol	1.67 ± 0.34	1.42 ± 0.26	1.70 ± 0.34	< 0.001
Triglycerides	0.75 ± 0.33	0.96 ± 0.44	0.73 ± 0.30	< 0.001
Glucose	4.88 ± 0.32	5.05 ± 0.33	4.86 ± 0.32	< 0.001
Waist perimeter	62.75 ± 8.49	75.46 ± 9.98	61.31 ± 6.98	< 0.001
Systolic blood pressure*	109.69 ± 11.96	117.46 ± 12.45	108.43 ± 11.40	< 0.001
Diastolic blood pressure*	65.07 ± 8.18	67.69 ± 8.32	64.65 ± 8.08	0.001

Data are presented as *N* (%) or the mean ± standard deviation. * denotes a total of 674 participants, with 94 in the MAFLD group and 580 in the non-MAFLD group. MAFLD, metabolic dysfunction-associated fatty liver disease

### Identification of alternative MAFLD biomarkers

**Fig. 2:**
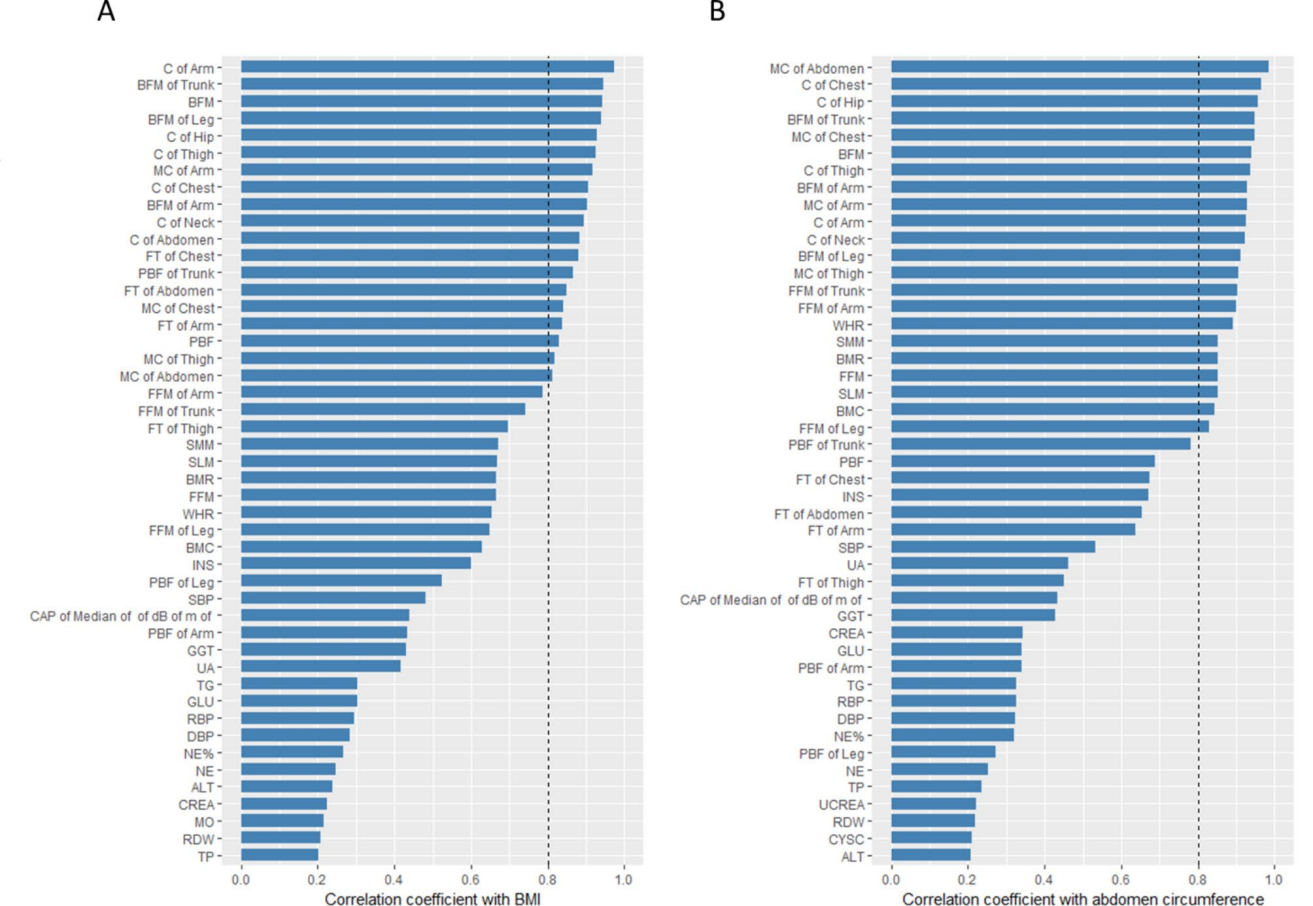
Correlations between MAFLD diagnostic biomarkers and other biomarkers. The bar graph displays the absolute values of the Pearson correlation coefficients (|*r*|) between biomarkers and BMI **(A)**, as well as biomarkers and abdomen circumference **(B)**. The dashed line represents a correlation coefficient threshold of 0.8, indicating a strong correlation. Biomarkers with *p-values* less than 0.05 or|*r*| values less than 0.2 are not shown

Alternative MAFLD biomarkers with strong and significant correlations (|*r*| > 0.8 and *p-value* < 0.05) with known MAFLD biomarkers are shown in Fig. 2. We found that PBF, the PBF of the trunk, and the thickness of fat in areas such as the chest, abdomen, and arms were strongly positively correlated with the diagnostic biomarker BMI. Similarly, for another known diagnostic biomarker waist circumference, we identified strongly positively correlated biomarkers including WHR, soft lean mass, basal metabolic rate, skeletal muscle mass, bone mineral content, fat-free mass (FFM), and specific regional FFM (e.g., arms, trunk, and legs). Notably, body fat mass (BFM), specific regional BFM (e.g., arms, trunk, and legs), neck, chest, hip, arm, and thigh circumferences, as well as muscle circumferences of the chest, abdomen, arms, and thighs, showed strong and positive correlations with BMI and waist perimeter. As shown in Table S4, among these factors, arm circumference presented the strongest correlation with BMI (*r* = 0.98, *p-value* < 0.001), whereas abdominal muscle circumference demonstrated the strongest association with waist perimeter (*r* = 0.99, *p-value* < 0.001).

### Identifying internal correlations among the remaining biomarkers

After these alternative biomarkers were excluded, the internal correlations (|*r*| > 0.8 and *p-value* < 0.05) among the remaining biomarkers (categorized by body composition, blood biochemistry, routine blood, and routine urine), are detailed in Tables S5 to S7.

Within the body composition dataset, notable correlations exist among the three biomarkers: the PBF of the leg, the fat thickness of the thigh, and the PBF of the arm. Specifically, the PBF of the leg exhibited the strongest correlation with the fat thickness of the thigh (*r* = 0.91, *p-value* < 0.001), followed by the considerable correlation observed between the PBF of the arm and the PBF of the leg (*r* = 0.87, *p-value* < 0.001). Furthermore, a relatively lower correlation was evident between the leg fat percentage and the chest fat percentage (*r* = 0.74, *p-value* < 0.001).

**Fig. 3:**
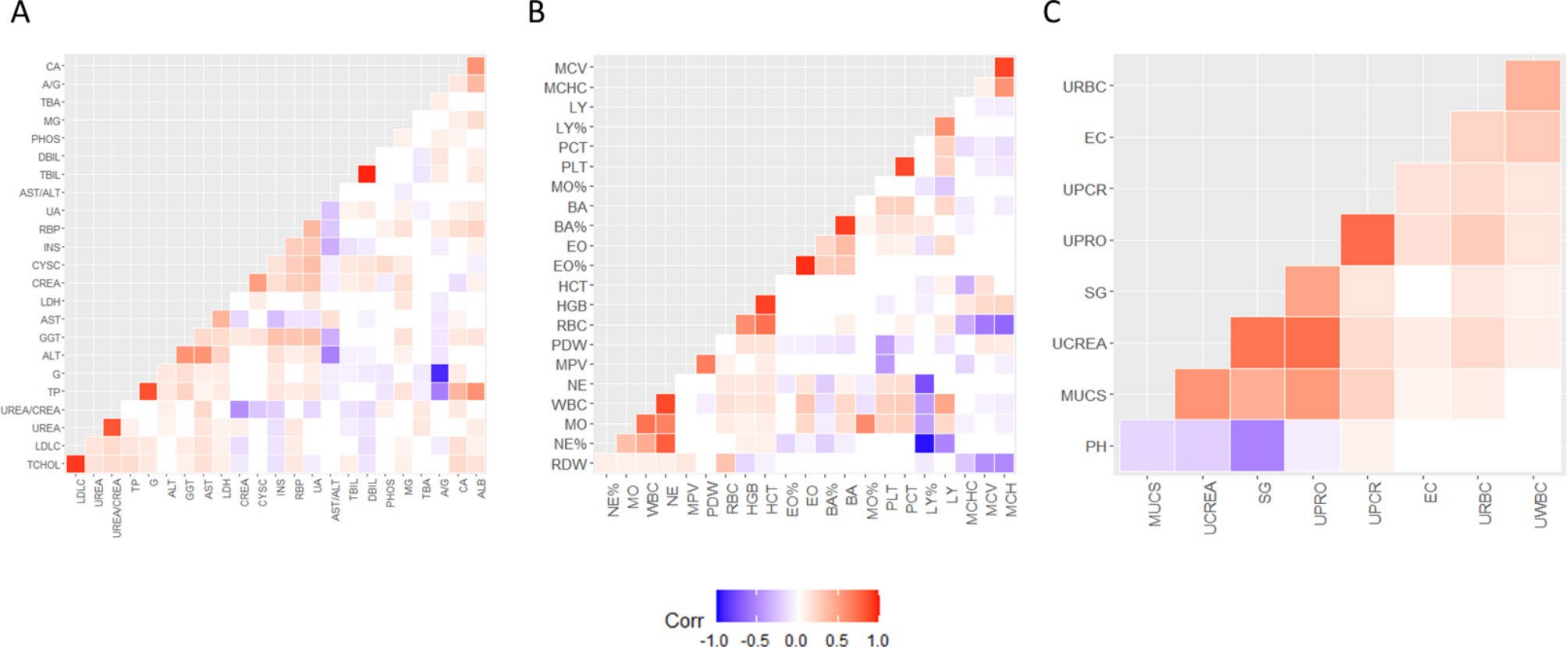
Correlation patterns within biomarkers. The heatmap displays the Pearson correlation coefficients (*r*) for blood biochemical biomarkers **(A)**, routine blood biomarkers **(B)**, and routine urine biomarkers **(C)** CA, calcium; G, globulin; TBA, total bile acid; Mg, magnesium; PHOS, phosphate; DBIL, direct bilirubin; TBIL, total bilirubin; AST, asparagine aminotransferase; ALT, alanine aminotransferase; UA, uric acid; RBP, retinol-binding protein; INS, insulin; CysC, cystatin C; CREA, creatinine; LDH, lactate dehydrogenase cholesterol; GGT, γ-glutamine transpeptidase; TP, total protein; LDHC, lactate dehydrogenase cholesterol; TCHOL, total cholesterol; MCV, mean corpuscular volume; MCHC, mean corpuscular hemoglobin concentration; LY, lymphocytes; PCT, platelet count; PLT, platelets; MO, monocyte; BA, basophil; EO, eosinophils; HCT, hematocrit; HGB, hemoglobin; RBC, red blood cell count; PDW, platelet distribution width; MPV, mean platelet volume; NE, neutrophils; WBC, white blood cells; RDW, red blood cell distribution width;; URBC, urine red blood cells; EC, epithelial cell count; UPCR, urinary protein creatinine ratio; UPRO, urine protein; SG, specific gravity; UCREA, urine creatinine; MUCS, Urine mucous strands; pH, potential of hydrogen

Figure 3A and Table S5 show that seven pairs of biomarkers were significantly correlated with the results of routine blood analysis: mean corpuscular volume and mean corpuscular hemoglobin (*r* = 0.88, *p-value* < 0.001), PLT and platelet count (PCT) (*r* = 0.87, *p-value* < 0.001), percentage of basophils and basophil count (*r* = 0.89, *p-value* < 0.001), percentage of eosinophil and eosinophil count (*r* = 0.94, *p-value* < 0.001), WBC and neutrophil count (*r* = 0.87, *p-value* < 0.001), percentage of lymphocytes (LY) and percentage of neutrophils (NE) (|*r*| = 0.97, *p-value* < 0.001), and hemoglobin and hematocrit (*r* = 0.89, *p-value* < 0.001). Conversely, Fig. 3B and Table S6 show that the results of the blood biochemical analysis revealed strong correlations among five pairs of biomarkers: LDLC and total cholesterol (*r* = 0.91, *p-value* < 0.001), the urea and urea/creatinine ratio (*r* = 0.82, *p-value* < 0.001), globulin and total protein (*r* = 0.84, *p-value* < 0.001), total bilirubin (TBIL) and direct bilirubin (*r* = 0.96, *p-value* < 0.001), and the globulin and albumin/globulin ratio (|*r*| = 0.91, *p-value* < 0.001). However, in the routine urinary analysis, no correlations were found among the biomarkers (as shown in Fig. 3C and Table S7).

### Identification of novel potential MAFLD biomarkers

The results from univariate logistic regression analysis, with age and sex as covariates, revealed that 29 variables were significantly associated with MAFLD. Specifically, creatinine, TBIL, direct bilirubin, AST/ALT, pH, percentage of LY, and percentage of eosinophils were negatively correlated with MAFLD, whereas the PBF of the leg, the PBF of the arm, the fat thickness of the thigh, calcium (Ca), WBC, insulin (INS), total protein, ALT, total bilirubin, lactate dehydrogenase, retinol-binding protein, uric acid, globulin, urine occult blood, urine specific gravity, percentage of NE, NE, monocyte, PLT, and PCT demonstrated a positive correlations with MAFLD (as shown in Table 2).

**Table 2:** Associations between biomarkers and MAFLD

Characteristics	Univariate logistic regression		Multivariate logistic regression	
	OR (95%CI)	*p*-value	OR (95%CI)	*p*-value
PBF of Arm	1.20 (1.15 to 1.24)	< 0.001	1.09 (1.01 to 1.19)	0.035
PBF of Leg	1.24 (1.19 to 1.29)	< 0.001	0.82 (0.72 to 0.94)	0.004
Fat Thickness of Thigh	2852.24 (729.29 to 11155.09)	< 0.001	11,500 (461.09 to 287000)	< 0.001
WBC	1.26 (1.13 to 1.40)	< 0.001		
NE%	1.04 (1.02 to 1.06)	< 0.001		
LY%	0.96 (0.94 to 0.99)	0.001		
EO%	0.88 (0.78 to 0.99)	0.028		
NE	1.29 (1.15 to 1.46)	< 0.001		
MO	23.71 (5.27 to 106.80)	< 0.001		
PLT	24,400 (604.96 to 991000)	< 0.001		
PCT	24481.18 (604.96 to 990694.57)	< 0.001	179.42 (1.79 to 17943.71)	0.027
Ca	1049.37 (60.37 to 18200)	< 0.001	66.37 (1.40 to 3153.33)	0.033
TBIL	0.85 (0.79 to 0.92)	< 0.001	0.91 (0.83 to 0.99)	0.038
DBIL	0.67 (0.54 to 0.82)	< 0.001		
TP	1.10 (1.04 to 1.17)	0.001		
ALT	1.07 (1.04 to 1.10)	< 0.001		
GGT	1.22 (1.15 to 1.30)	< 0.001		
LDH	1.01 (1.00 to 1.01)	0.003		
CREA	0.95 (0.91 to 0.99)	0.006		
RBP	1.07 (1.03 to 1.10)	< 0.001		
UA	1.01 (1.00 to 1.01)	< 0.001		
G	1.11 (1.03 to 1.19)	0.004		
AST/ALT	0.10 (0.06 to 0.17)	< 0.001	0.27 (0.14 to 0.49)	< 0.001
INS	1.27 (1.20 to 1.33)	< 0.001	1.09 (1.03 to 1.16)	0.006
BLD	1.56 (1.02 to 2.39)	< 0.001		
USG	2.27 × 10^30^ (1.90 × 10^13^ to 2.72 × 10^47^)	< 0.001		
pH	0.47 (0.32 to 0.70)	< 0.001	0.46 (0.28 to 0.78)	0.004

Associations between MAFLD and these variables were measured separately, with age and sex considered as covariates. Variables with *p-value* > 0.05 are not shown. MAFLD, metabolic dysfunction-associated fatty liver disease; PBF, percent body fat; WBC, white blood cell; NE, neutrophils; LY, lymphocyte; EO, eosinophils; MO, monocyte; PLT, platelet; PCT, platelet count; Ca, calcium; TBIL, total bilirubin; DBIL, direct bilirubin; TP, total protein; ALT, alanine aminotransferase; GGT, γ-glutamine transpeptidase; LDH, lactate dehydrogenase; CREA, creatinine; RBP, retinol-binding protein; UA, uric acid; G, globulin; AST, asparagine aminotransferase; INS, insulin; BLD, urine occult blood; USG, urine specific gravity; pH, potential of hydrogen; %, percentage; /, ratio

**Fig. 4:**
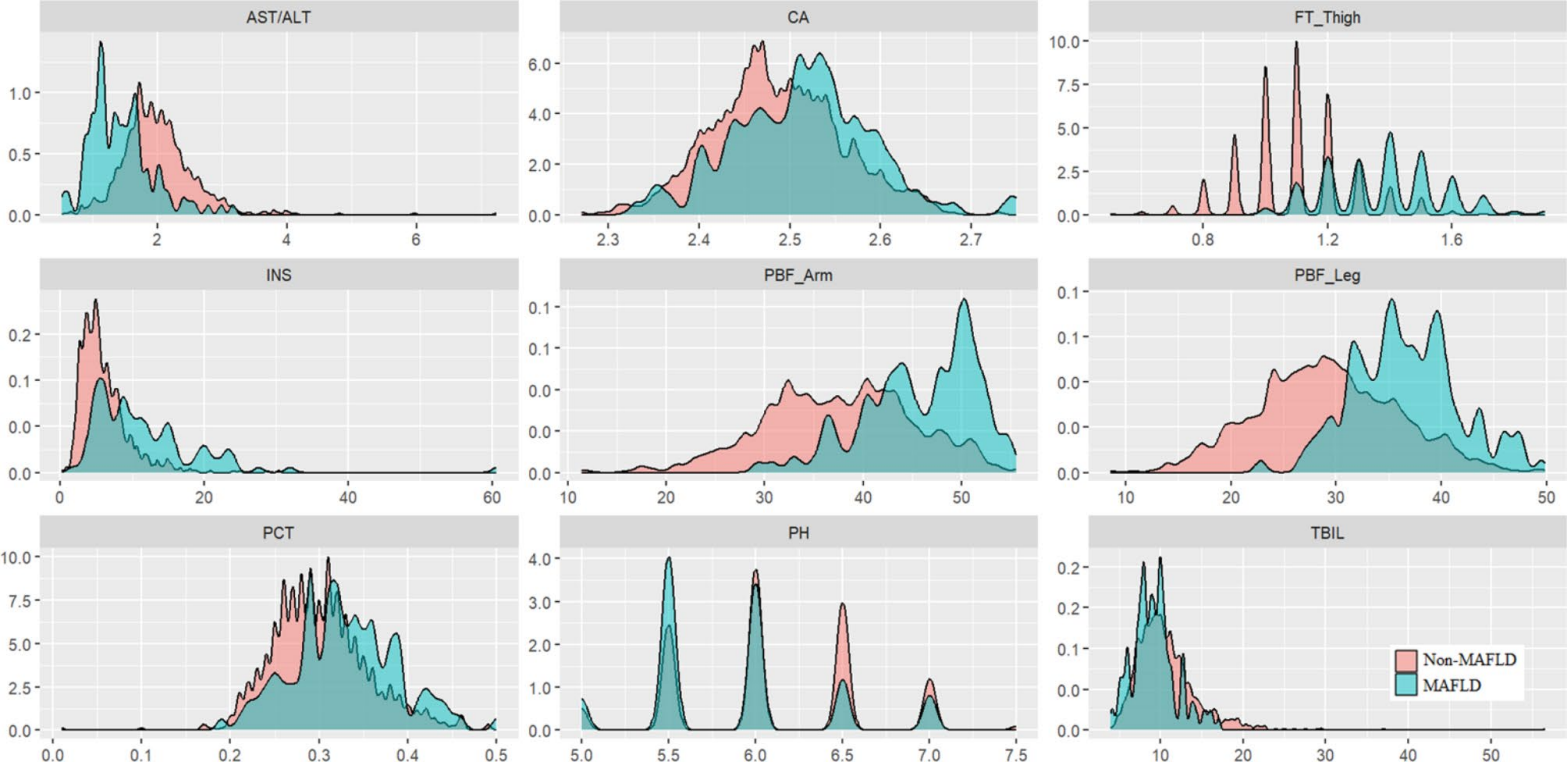
Distribution of novel potential MAFLD biomarkers. The density plot displays the distribution of novel potential biomarkers in the MAFLD (red) and non-MAFLD (blue) groups. AST, asparagine aminotransferase; ALT, alanine aminotransferase; CA, calcium; FT, fat thickness; PBF, Percent Body Fat; INS, insulin; PCT, platelet count; pH, potential of hydrogen; TBIL, total bilirubin

By performing multivariate stepwise logistic regression analysis with age and sex as covariates, 9 biomarkers were ultimately determined to be potential novel potential MAFLD biomarkers. As shown in Table 2, these biomarkers included the 5 blood-based biomarkers: AST/ALT ratio (OR = 0.27, 95% CI = 0.14–0.49), TBIL (OR = 0.91, 95% CI = 0.83–0.99), PCT (OR = 179.42, 95% CI = 1.79-17943.71), Ca (OR = 66.37, 95% CI = 1.40-3153.33), INS (OR = 1.09, 95% CI = 1.03–1.16); 3 body component measurements: PBF of the leg (OR = 0.82, 95% CI = 0.72–0.94), PBF of the arm (OR = 1.09, 95% CI = 1.01–1.19), fat thickness of the thigh (OR = 11500, 95% CI = 461.09-287000); and urine pH (OR = 0.46, 95% CI = 0.28–0.78). The distributions of these biomarkers between MAFLD and non-MAFLD patients are depicted in Fig. 4.

## Discussion

In this cross-sectional study, a comprehensive analysis of blood, urine, and body composition biomarkers was conducted to assess the relationships between less-explored composition biomarkers and MAFLD. This study revealed that all biomarkers in body composition were closely associated with MAFLD, and could serve as alternative or novel potential biomarkers. These findings emphasize the importance of body composition testing, and focusing on these biomarkers can aid in the early identification of MAFLD patients.

The prevalence of MAFLD in children in this study was 10.2%. This value was slightly higher than that reported in a cohort study conducted with 1350 school children in China, which reported a prevalence of 7.0% for MAFLD (7.5% in males, 2.5% in females) [31]. Furthermore, consistent with earlier research, MAFLD was more common in males than in females [18, 32–34]. This discrepancy primarily arises from the hormonal regulatory variances between males and females. Oestrogen plays a role in regulating blood lipid metabolism and intrahepatic fat distribution. This includes its capacity to inhibit the accumulation of visceral fat and promote the deposition of subcutaneous fat, potentially enhancing MAFLD [35–37]. Adiponectin, a protein secreted by adipocytes, has the capacity to inhibit liver inflammation and delay liver tissue fibrosis [38]. Nishizawa reported that androgens can lower plasma adiponectin concentrations [39]. In men, higher androgen concentrations result in lower plasma adiponectin concentrations, which leads to a higher incidence of MAFLD.

This study clearly identified alternative biomarkers that were correlated with highly relevant biomarkers for diagnosis (i.e., all body composition biomarkers except the PBF of the arm, the PBF of the leg, and the thickness of the thigh). Notably, these alternative biomarkers are significantly correlated with obesity, a connection that has been widely confirmed by research. While research regarding the correlation between PBF and MAFLD remains limited, studies have suggested that PBF may better predict obesity than BMI [40–42]. Furthermore, the WHR serves as a crucial indicator for evaluating central obesity, effectively depicting the distribution of abdominal visceral fat and subcutaneous fat. Studies by Zheng et al. [43]., Cai et al. [44]., and Zhou et al. [45] have shown its significant diagnostic value for MAFLD. These findings underscore the potential association between MAFLD occurrence and the distribution of central body fat. Additionally, biomarkers such as chest circumference, neck circumference, hip circumference, and soft lean mass have been validated as MAFLD related biomarkers [46, 47]. Therefore, the alternative biomarkers identified in this study have a certain utility in screening for MAFLD and its prevention in children. These body measurement parameters are easily obtained during routine health examinations. Focusing on body composition test data in children will provide pediatricians with convenient preliminary guidance, aiding in routine health assessments for children.

After sex and age were considered as covariates, novel potential biomarkers were explored, and found to be correlated with MAFLD but unrelated to diagnostic biomarkers (i.e., the PBF of the arm, the PBF of the leg, the thickness of the thigh, PCT, Ca, TBIL, INS, pH, and AST/ALT). Compared with healthy participants, patients with MAFLD presented significantly elevated concentrations of PCT, Ca, the PBF of the arm, the PBF of the leg, and thickness of the thigh. Although these biomarkers do not have a direct relationship with MAFLD diagnosis, their elevation may reflect changes in the metabolism and fat distribution of MAFLD patients. These findings provide a new perspective for studying biomarkers in the early stages of MAFLD and offer potential clues for early intervention and treatment.

Currently, research on the relationships among blood calcium, urine pH, and MAFLD is relatively limited. This study revealed a positive correlation between Ca concentrations and MAFLD, which is consistent with the findings of a cross-sectional study conducted in South Korea [48]. However, there is still controversy regarding the relationship between blood Ca and MAFLD. Some studies suggest that a decrease in blood Ca concentrations may increase the risk of developing MAFLD, whereas others argue that there is no association between the two [49–51]. This inconsistency may stem from differences in ethnicity, study design, or concentration measurement methods. Furthermore, our findings align with those of the only two existing studies, which demonstrated a negative correlation between urine pH and MAFLD [52, 53]. Both of these factors, as well as the interaction between INS and MAFLD, are associated with insulin resistance, which has been widely confirmed as a predictor of MAFLD [42–44]. The primary reasons include compromised fat breakdown, increased transport of free fatty acids to the liver, and enhanced new fat synthesis, resulting in hepatic steatosis [54].

Research suggests a significant negative correlation between fibrosis stage and platelet count [55]. PCT, which serves as an indicator of the PLT, has been proven to be an effective parameter for predicting significant or advanced fibrosis and cirrhosis, demonstrating fairly high diagnostic accuracy [56]. Additionally, markers associated with liver fibrosis include AST/ALT; the 2018 edition of China’s MAFLD treatment guidelines indicate that elevated concentrations of ALT, AST, and other biomarkers significantly increase the incidence of MAFLD and MASH [57]. MAFLD encompasses a spectrum starting from basic fatty liver to metabolic dysfunction associated with steatohepatitis, progressing towards advanced fibrosis and eventual cirrhosis [58]. Therefore, early identification of fibrosis and cirrhosis in MAFLD patients is of paramount importance for preventing complications.

Notably, the PBF of the arm was positively correlated with MAFLD according to univariate logistic regression analysis; however, in multivariate stepwise logistic regression analysis, revealed a negative correlation. Additionally, significant differences were observed in the effect values of PCT, CA, and the fat thickness of the thigh between univariate and multivariate stepwise logistic regression analyses. The likely reason for this variance is that the multivariate analysis fully considers confounding factors by integrating all variables selected in the univariate analysis. The presence of collinearity, stemming from the interdependence of these variables, has influenced the interpretation of their respective contributions to MAFLD. The Pearson correlation analysis results also confirmed the high correlation between these variables, introducing complexity to the explanation of differences in the effects in the multivariate stepwise logistic analysis. However, in addition to the covariates that were adjusted for in the present study, several confounding factors, including lifestyle, genetics, and the environment, have previously been reported to be linked to MAFLD [59–61]. The absence of these factors in this study hinders further analysis of these confounding factors. Therefore, future research should explore the intricate relationships between these biomarkers and confounding factors to comprehensively understand their roles in the progression of MAFLD.

This study’s strength lies in its extensive analysis of MAFLD-related biomarkers among diverse pediatric populations. Nevertheless, it is crucial to acknowledge that this is an exploratory analysis with several potential limitations. First, owing to challenges in recruiting pediatric patients from multiple regions, sample selection bias may impact the external validity of the research results. Second, given the impracticality of conducting widespread liver biopsies, the study diagnosed MAFLD using FibroScan examinations to identify hepatic steatosis, which may affect diagnostic accuracy. Furthermore, excluding certain patients due to missing data and not using age- and sex-adjusted measurements (such as Z scores and percentiles) when studying body composition biomarkers may introduce bias. Last, stepwise logistic regression analysis carries the risk of model overfitting, and interactions among other factors may affect the interpretation of the study results.

## Conclusion

This study conducted a comprehensive analysis aiming to evaluate the MAFLD related biomarkers in children undergoing physical examinations. The findings revealed an overall prevalence of MAFLD in children of 10.2% (14.99% in boys, 5.18% in girls). Significantly, factors such as the PBF of the leg, the PBF of the arm, the fat thickness of the thigh, the pH, TBIL, Ca, INS, PCT concentrations and the AST/ALT ratio were identified as biomarkers related to MAFLD among children. Additionally, apart from the PBF of the leg, the PBF of the arm, and the fat thickness of the thigh, all other body composition biomarkers were significantly correlated with the BMI or waist perimeter in the MAFLD diagnostic criteria, demonstrating their potential use as alternative biomarkers. These findings serve as a reference for MAFLD management protocols among the Chinese paediatric population, helping healthcare professionals (e.g., physicians, nurses, nutritionists, etc.) achieve early detection, early detection, and early intervention for children at high-risk of MAFLD.

## Electronic supplementary material

Below is the link to the electronic supplementary material.

Supplementary Material 1

## Data Availability

You can find the data available at https://jinqiao-cohort.readthedocs.io/en/latest/.

## Abbreviations

MAFLD: Metabolic associated fatty liver
T2DM: Type 2 diabetes mellitus
HDLC: High-density lipoprotein cholesterol
ALT: Alanine aminotransferase
GLU: Glucose
WHR: Waist-to-hip ratio
TG: Triglyceride
AST: Aspartate aminotransferase
LDLC: Low-density lipoprotein cholesterol
CAP: Controlled attenuation parameter
SBP: Systolic blood pressure
DBP: Diastolic blood pressure
BMI: Body mass index
WBC: White blood cell
PLT: Platelet
pH: Potential of hydrogen
SD: Standard deviation
FFM: Fat-free mass
BFM: Body fat mass
PCT: Platelet count
LY: Lymphocyte
NE: Neutrophil
TBIL: Total bilirubin
Ca: Calcium
INS: Insulin
