# Supplementary material for: Potential alternative and novel biomarkers for paediatric MAFLD: exploratory evidence from a Chinese cohort
Source: BMC Gastroenterol. 2025 Jan 22;25:28. doi: 10.1186/s12876-025-03619-2 (PMC11752933; doi:10.1186/s12876-025-03619-2)
Supplement: Supplementary file 1 — Supplementary Material 1 [file 12876_2025_3619_MOESM1_ESM.docx]

**Table S1. Laboratory examination items.**

| **Abbreviation** | **Normal range** | **Unit** |
| --- | --- | --- |
| Red blood cell count | NA | 10^12^/L |
| Hemoglobin | NA | g/L |
| Hematocrit | NA | % |
| Mean corpuscular volume | 80-100 | fL |
| Mean corpuscular hemoglobin | 27-34 | pg |
| Mean corpuscular hemoglobin concentration | 316-354 | g/L |
| Red blood cell distribution width | 11.5-14.9 | % |
| White blood cells | 3.5-9.5 | 10^19^/L |
| Neutrophils % | 40-75 | % |
| Neutrophils | 1.8-6.3 | 10^9^/L |
| Lymphocytes % | 20-50 | % |
| Lymphocytes | 1.1-3.2 | 10^9^/L |
| Monocyte % | 3-10 | % |
| Monocyte | 0.1-0.6 | 10^9^/L |
| Eosinophils % | 0.4-8 | % |
| Eosinophils | 0.02-0.52 | 10^9^/L |
| Basophil % | 0-1 | % |
| Basophil | 0-0.06 | 10^9^/L |
| Platelets | 125-350 | 10^9^/L |
| Mean platelet volume | 6-11.5 | fL |
| Platelet distribution width | 9-17 | fL |
| Platelet count | 0.108-0.272 | fL |
| Triglycerides | <1.7 | mmol/L |
| Total cholesterol | <5.18 | mmol/L |
| Lactate dehydrogenase cholesterol | <3.37 | mmol/L |
| High-density lipoprotein cholesterol | NA | mmol/L |
| Albumin | 40-55 | g/L |
| Globulin | 20-40 | g/L |
| uric acid / Globulin | 1.20-2.40 | NA |
| Asparagine aminotransferase | NA | U/L |
| Alanine aminotransferase | NA | U/L |
| Asparagine aminotransferase / Alanine aminotransferase aminotransferase | NA | NA |
| γ-glutamine transpeptidase | NA | U/L |
| Total bilirubin | NA | umol/L |
| Directbilirubin | 0-6 | umol/L |
| Total protein | 65-85 | g/L |
| Lactate dehydrogenase | 120-250 | U/L |
| Total bile acid | 0-10 | umol/L |
| Retinol-binding protein | 25-70 | mg/L |
| Uric acid | NA | umol/L |
| Glucose | 3.9-6.1 | mmol/L |
| Insulin | NA | mU/L |
| Urea | NA | mmol/L |
| Creatinine | NA | umol/L |
| Cystatin C | 0.6-1.5 | mg/L |
| Urea/ Creatinine | NA | NA |
| Calcium | 2.2-2.7 | mmol/L |
| Phosphate | 0.85-1.51 | mmol/L |
| Magnesium | 0.75-1.02 | mmol/L |
| Urine specific gravity | 1.010-1.025 | NA |
| Potential of hydrogen | 5.5-7.0 | NA |
| Urine occult blood | NA | NA |
| Urinary calcium | 1.5-9.0 | mmol/L |
| Urine red blood cells | 0-10 | ul |
| Urine white blood cells | 0-10 | ul |
| Epithelial cell count | 0-10 | ul |
| Urine mucous strands | 0-15 | ul |
| Urine creatinine | NA | umol/L |
| Vitamin C | NA | NA |
| Leukocyte esterase | NA | NA |
| Urobilinogen | NA | NA |
| Protein | NA | NA |
| Urine protein | 0.00-0.15 | g/L |

%, percentage; /, ratio.

**Table S2.** Baseline characteristics of the participants.

| **Characteristics** | | **All (*N*=1108)** | **MAFLD (*N*=113)** | **Non-MAFLD (*N*=995)** | ***p-value*** |
| --- | --- | --- | --- | --- | --- |
| Body composition biomarkers | Body Fat Mass | 9.45 ± 5.72 | 18.78 ± 6.28 | 8.39 ± 4.57 | <0.001 |
|  | Soft Lean Mass | 25.64 ± 6.78 | 31.78 ± 6.94 | 24.95 ± 6.40 | <0.001 |
|  | Fat Free Mass | 27.29 ± 7.24 | 33.82 ± 7.41 | 26.54 ± 6.84 | <0.001 |
|  | Skeletal Muscle Mass | 14.06 ± 4.25 | 17.94 ± 4.34 | 13.62 ± 4.01 | <0.001 |
|  | Percent Body Fat | 24.19 ± 7.78 | 34.88 ± 6.14 | 22.98 ± 6.98 | <0.001 |
|  | Basal Metabolic Rate | 959.33 ± 156.38 | 1100.52 ± 160.08 | 943.29 ± 147.72 | <0.001 |
|  | Waist-Hip Ratio | 0.77 ± 0.04 | 0.82 ± 0.05 | 0.76 ± 0.03 | <0.001 |
|  | Bone Mineral Content | 1.64 ± 0.46 | 2.04 ± 0.48 | 1.60 ± 0.44 | <0.001 |
|  | Fat Free Mass of Arm | 1.02 ± 0.41 | 1.48 ± 0.44 | 0.97 ± 0.37 | <0.001 |
|  | Fat Free Mass of Trunk | 11.31 ± 3.17 | 14.65 ± 3.25 | 10.93 ± 2.94 | <0.001 |
|  | Fat Free Mass of Leg | 3.74 ± 1.35 | 4.93 ± 1.37 | 3.60 ± 1.28 | <0.001 |
|  | Body Fat Mass of Arm | 0.71 ± 0.42 | 1.40 ± 0.55 | 0.63 ± 0.31 | <0.001 |
|  | Body Fat Mass of Trunk | 3.78 ± 3.17 | 8.89 ± 3.37 | 3.20 ± 2.57 | <0.001 |
|  | Body Fat Mass of Leg | 1.71 ± 0.82 | 3.05 ± 0.89 | 1.56 ± 0.66 | <0.001 |
|  | Percent Body Fat of Arm | 38.39 ± 7.77 | 45.83 ± 5.71 | 37.55 ± 7.52 | <0.001 |
|  | Percent Body Fat of Trunk | 20.64 ± 10.76 | 34.85 ± 7.84 | 19.03 ± 9.83 | <0.001 |
|  | Percent Body Fat of Leg | 29.86 ± 6.92 | 36.63 ± 5.07 | 29.10 ± 6.68 | <0.001 |
|  | Circumference of Neck | 9.47 ± 1.80 | 30.15 ± 2.97 | 25.58 ± 2.67 | <0.001 |
|  | Circumference of Chest | 71.94 ± 8.19 | 83.11 ± 7.92 | 70.67 ± 7.20 | <0.001 |
|  | Circumference of Hip | 81.43 ± 7.14 | 91.61 ± 6.97 | 80.28 ± 6.18 | <0.001 |
|  | Circumference of Arm | 22.75 ± 3.12 | 27.39 ± 2.84 | 22.22 ± 2.69 | <0.001 |
|  | Circumference of Thigh | 43.19 ± 4.55 | 49.69 ± 4.46 | 42.46 ± 3.94 | <0.001 |
|  | Muscle Circumference of Chest | 67.56 ± 7.35 | 76.57 ± 6.94 | 66.53 ± 6.67 | <0.001 |
|  | Muscle Circumference of Abdomen | 57.57 ± 7.53 | 67.79 ± 8.85 | 56.41 ± 6.41 | <0.001 |
|  | Muscle Circumference of Arm | 19.14 ± 2.51 | 22.46 ± 2.19 | 18.76 ± 2.26 | <0.001 |
|  | Muscle Circumference of Thigh | 36.14 ± 4.11 | 41.02 ± 3.87 | 35.59 ± 3.75 | <0.001 |
|  | Fat Thickness of Chest | 0.70 ± 0.22 | 1.05 ± 0.22 | 0.66 ± 0.19 | <0.001 |
|  | Fat Thickness of Abdomen | 0.82 ± 0.26 | 1.22 ± 0.25 | 0.78 ± 0.22 | <0.001 |
|  | Fat Thickness of Arm | 0.58 ± 0.14 | 0.78 ± 0.14 | 0.55 ± 0.12 | <0.001 |
|  | Fat Thickness of Thigh | 1.12 ± 0.20 | 1.38 ± 0.18 | 1.09 ± 0.17 | <0.001 |
| Blood biomarkers | Insulin | 6.52 ± 4.34 | 11.54 ± 7.66 | 5.96 ± 3.35 | <0.001 |
|  | Ca | 2.49 ± 0.07 | 2.51 ± 0.08 | 2.49 ± 0.07 | <0.001 |
|  | Phosphate | 1.69 ± 0.13 | 1.68 ± 0.13 | 1.69 ± 0.13 | 0.384 |
|  | Magnesium | 0.84 ± 0.05 | 0.85 ± 0.05 | 0.84 ± 0.05 | 0.059 |
|  | Total bilirubin | 10.56 ± 3.84 | 9.24 ± 2.63 | 10.71 ± 3.93 | <0.001 |
|  | Direct bilirubin | 3.75 ± 1.26 | 3.38 ± 0.95 | 3.79 ± 1.28 | <0.001 |
|  | Total protein | 75.27 ± 3.37 | 76.47 ± 3.41 | 75.13 ± 3.34 | <0.001 |
|  | Albumin | 45.77 ± 1.82 | 46.20 ± 1.47 | 45.72 ± 1.85 | 0.007 |
|  | Alanine aminotransferase | 14.50 ± 7.76 | 19.90 ± 14.85 | 13.88 ± 6.21 | <0.001 |
|  | γ-glutamine transpeptidase | 13.75 ± 3.80 | 17.28 ± 7.56 | 13.35 ± 2.85 | <0.001 |
|  | Asparagine aminotransferase | 25.71 ± 5.68 | 24.56 ± 6.56 | 25.84 ± 5.56 | <0.001 |
|  | Lactate dehydrogenase | 234.38 ± 37.04 | 248.03 ± 38.03 | 232.83 ± 36.62 | <0.001 |
|  | Total bile acid | 2.88 ± 2.04 | 3.07 ± 2.01 | 2.86 ± 2.04 | 0.270 |
|  | Urea | 4.85 ± 1.01 | 4.92 ± 0.98 | 4.84 ± 1.01 | 0.402 |
|  | Creatinine | 44.93 ± 6.10 | 45.28 ± 5.69 | 44.89 ± 6.14 | 0.418 |
|  | Cystatin C | 0.77 ± 0.08 | 0.79 ± 0.08 | 0.77 ± 0.08 | 0.003 |
|  | Retinol-binding protein | 33.93 ± 5.85 | 36.68 ± 5.79 | 33.61 ± 5.78 | <0.001 |
|  | Uric acid | 296.10 ± 64.26 | 341.04 ± 72.45 | 290.99 ± 61.25 | <0.001 |
|  | Globulin | 29.50 ± 2.79 | 30.27 ± 3.12 | 29.42 ± 2.74 | 0.011 |
|  | Asparagine aminotransferase / Alanine aminotransferase | 1.94 ± 0.58 | 1.43 ± 0.49 | 2.00 ± 0.56 | <0.001 |
|  | Uric acid / Globulin | 1.57 ± 0.16 | 1.54 ± 0.17 | 1.57 ± 0.16 | 0.157 |
|  | Urea/ Creatinine | 0.11 ± 0.03 | 0.11 ± 0.02 | 0.11 ± 0.03 | 0.822 |
|  | Total cholesterol | 4.72 ± 0.84 | 4.60 ± 0.82 | 4.73 ± 0.84 | 0.183 |
|  | Lactate dehydrogenase cholesterol | 2.27 ± 0.60 | 2.25 ± 0.62 | 2.28 ± 0.60 | 0.825 |
|  | White blood cells | 6.64 ± 1.61 | 7.34 ± 1.73 | 6.56 ± 1.57 | <0.001 |
|  | Neutrophils% | 46.98 ± 9.55 | 50.83 ± 8.21 | 46.54 ± 9.60 | <0.001 |
|  | Monocyte% | 5.56 ± 1.24 | 5.73 ± 1.15 | 5.54 ± 1.25 | 0.043 |
|  | Lymphocyte% | 44.16 ± 9.43 | 40.35 ± 7.96 | 44.59 ± 9.49 | <0.001 |
|  | Eosinophils% | 2.92 ± 1.95 | 2.73 ± 1.38 | 2.94 ± 2.01 | 0.775 |
|  | Basophil% | 0.38 ± 0.21 | 0.37 ± 0.21 | 0.39 ± 0.21 | 0.367 |
|  | Neutrophils | 3.19 ± 1.33 | 3.81 ± 1.38 | 3.12 ± 1.30 | <0.001 |
|  | Monocyte | 0.37 ± 0.11 | 0.42 ± 0.12 | 0.36 ± 0.11 | <0.001 |
|  | Lymphocytes | 2.87 ± 0.71 | 2.89 ± 0.62 | 2.87 ± 0.72 | 0.554 |
|  | Eosinophils | 0.19 ± 0.14 | 0.20 ± 0.12 | 0.19 ± 0.14 | 0.065 |
|  | Basophil | 0.02 ± 0.01 | 0.03 ± 0.02 | 0.02 ± 0.01 | 0.267 |
|  | Red blood cell count | 4.80 ± 0.32 | 4.86 ± 0.34 | 4.80 ± 0.32 | 0.057 |
|  | Hemoglobin | 135.01 ± 6.94 | 135.65 ± 7.83 | 134.93 ± 6.83 | 0.378 |
|  | Hematocrit | 41.11 ± 2.24 | 41.36 ± 2.45 | 41.08 ± 2.21 | 0.316 |
|  | Mean corpuscular volume | 85.71 ± 3.76 | 85.24 ± 3.28 | 85.76 ± 3.81 | 0.082 |
|  | Mean corpuscular hemoglobin | 28.16 ± 1.45 | 27.97 ± 1.33 | 28.18 ± 1.46 | 0.044 |
|  | Mean corpuscular hemoglobin concentration | 328.56 ± 8.21 | 328.09 ± 8.74 | 328.61 ± 8.15 | 0.513 |
|  | Platelet distribution width | 12.98 ± 0.58 | 13.14 ± 0.55 | 12.96 ± 0.58 | <0.001 |
|  | Platelets | 305.21 ± 62.13 | 327.19 ± 66.19 | 302.71 ± 61.19 | <0.001 |
|  | Platelet count | 0.30 ± 0.06 | 0.33 ± 0.06 | 0.30 ± 0.05 | <0.001 |
|  | Mean platelet volume | 10.05 ± 1.03 | 10.11 ± 0.99 | 10.05 ± 1.04 | 0.308 |
|  | Platelet distribution width | 15.92 ± 0.30 | 15.93 ± 0.28 | 15.92 ± 0.30 | 0.545 |
| Urine biomarkers | Urine occult blood |  |  |  | 0.114 |
|  | - | 1030(92.96%) | 100(88.50%) | 930(93.47%) |  |
|  | ± | 62(5.60%) | 10(8.85%) | 52(5.23%) |  |
|  | 1+ | 13(1.17%) | 3(2.65%) | 10(1.01%) |  |
|  | 3+ | 3(0.27%) | 3(2.59%) | 3(0.30%) |  |
|  | Urobilinogen |  |  |  | 0.513 |
|  | - | 1079(97.38%) | 109(96.46%) | 970(97.49%) |  |
|  | 1+ | 26(2.35%) | 4(3.54%) | 22(2.21%) |  |
|  | 2+ | 3(0.27%) | 0(0.00%) | 3(0.30%) |  |
|  | Protein |  |  |  | 1.000 |
|  | - | 1081(97.83%) | 111(98.23%) | 970(97.78%) |  |
|  | ± | 13(1.18%) | 1(0.88%) | 12(1.21%) |  |
|  | 1+ | 11(1.00%) | 1(0.88%) | 10(1.01%) |  |
|  | Vitamin C |  |  |  | 0.348 |
|  | - | 927(83.66%) | 98(86.73%) | 829(83.32%) |  |
|  | ± | 59(5.32%) | 2(1.77%) | 57(5.73%) |  |
|  | 1+ | 41(3.70%) | 4(3.54%) | 37(3.72%) |  |
|  | 2+ | 31(2.80%) | 2(1.77%) | 29(2.91%) |  |
|  | 3+ | 50(4.51%) | 7(6.19%) | 43(4.32%) |  |
|  | Leukocyte esterase |  |  |  | 0.017 |
|  | - | 790(71.30%) | 96(84.96%) | 694(69.75%) |  |
|  | ± | 148(13.36%) | 8(7.08%) | 140(14.07%) |  |
|  | 1+ | 90(8.12%) | 4(3.54%) | 86(8.64%) |  |
|  | 2+ | 50(4.51%) | 3(2.65%) | 47(4.72%) |  |
|  | 3+ | 30(2.71%) | 2(1.77%) | 28(2.81%) |  |
|  | Urinary calcium |  |  |  | 0.002 |
|  | <1.00 | 139(12.55%) | 26(23.01%) | 26(23.01%) |  |
|  | 2.5 | 770(69.49%) | 65(57.52%) | 65(57.52%) |  |
|  | 5 | 199(17.96%) | 22(19.47%) | 22(19.47%) |  |
|  | Urine red blood cells | 2.60 ± 4.76 | 2.89 ± 5.27 | 2.57 ± 4.69 | 0.971 |
|  | Urine white blood cells | 8.83 ± 22.99 | 7.38 ± 19.61 | 8.99 ± 23.35 | 0.065 |
|  | Urine protein | 0.09 ± 0.07 | 0.09 ± 0.06 | 0.09 ± 0.07 | 0.026 |
|  | Urinary protein creatinine ratio | 0.07 ± 0.03 | 0.07±0.03 | 0.07 ± 0.03 | 0.012 |
|  | Urine creatinine | 10559.69 ± 4679.35 | 12004.09 ± 4460.43 | 10395.65 ± 4677.57 | <0.001 |
|  | Urine specific gravity | 1.02 ± 0.01 | 1.02 ± 0.01 | 1.02 ± 0.01 | <0.001 |
|  | Potential of hydrogen | 6.08 ± 0.54 | 5.87 ± 0.51 | 6.10 ± 0.53 | <0.001 |
|  | Epithelial cell count | 3.54 ± 10.87 | 3.62 ± 14.09 | 3.53 ± 10.45 | 0.471 |
|  | Urine mucous strands | 25.88 ± 52.01 | 26.55 ± 34.63 | 25.80 ± 53.64 | 0.257 |

MAFLD, metabolic dysfunction-associated fatty liver disease; %, percentage; /, ratio.

**Table S3.** Baseline characteristics of participants stratified by sex.

| **Characteristics** | **Male** | | | | **Female** | | | |  |
| --- | --- | --- | --- | --- | --- | --- | --- | --- | --- |
|  |  |  |  |  |  |  |  |  |  |
|  | **All**  **(*N*=567)** | **MAFLD**  **(*N*=85)** | **Non-MAFLD**  **(*N*=482)** | ***p-value*** | **All**  **(*N*=541)** | **MAFLD**  **(*N*=28)** | **Non-MAFLD**  **(*N*=513)** | ***p-value*** |  |
|  |  |  |  |  |  |  |  |  |  |
| Age | 9.55 ± 1.76 | 9.39 ± 1.80 | 10.42 ± 1.44 | <0.001 | 9.40 ± 1.82 | 9.36 ± 1.81 | 10.16 ± 1.72 | 0.020 |  |
| Body mass index Z-score | 0.35 ± 1.53 | 0.65 ± 1.58 | 0.30 ± 1.52 | 0.055 | 0.32 ± 1.46 | 0.79 ± 1.85 | 0.29 ± 1.44 | 0.186 |  |
| High-density lipoprotein cholesterol | 1.66 ± 0.35 | 1.70 ± 0.34 | 1.43 ± 0.28 | <0.001 | 1.69 ± 0.33 | 1.71 ± 0.33 | 1.41 ± 0.22 | <0.001 |  |
| Triglycerides | 0.74 ± 0.36 | 0.70 ± 0.33 | 0.93 ± 0.45 | <0.001 | 0.77 ± 0.29 | 0.75 ± 0.27 | 1.04 ± 0.40 | <0.001 |  |
| Glucose | 4.93 ± 0.30 | 4.91 ± 0.30 | 5.04 ± 0.28 | <0.001 | 4.83 ± 0.34 | 4.81 ± 0.32 | 5.07 ± 0.44 | 0.004 |  |
| Waist Circumference | 6.92 ± 9.38 | 61.75 ± 7.37 | 76.24 ± 10.09 | <0.001 | 61.52 ± 7.25 | 60.89 ± 6.57 | 73.07 ± 9.41 | <0.001 |  |
| Systolic blood pressure | 110.93 ± 12.32 | 117.45 ± 12.89 | 109.24 ± 11.61 | <0.001 | 108.31 ± 11.41 | 117.48 ± 11.05 | 107.67 ± 11.17 | <0.001 |  |
| Diastolic blood pressure | 65.04 ± 8.46 | 67.74 ± 8.39 | 64.34 ± 8.35 | 0.001 | 65.10 ± 7.87 | 67.52 ± 8.28 | 64.93 ± 7.82 | 0.240 |  |
| Body Fat Mass | 10.29 ± 6.47 | 19.31 ± 6.36 | 8.70 ± 5.03 | <0.001 | 8.58 ± 4.65 | 17.19 ± 5.88 | 8.11 ± 4.08 | <0.001 |  |
| Soft Lean Mass | 26.73 ± 7.11 | 32.26 ± 6.84 | 25.75 ± 6.71 | <0.001 | 24.50 ± 6.22 | 30.30 ± 7.17 | 24.19 ± 6.01 | <0.001 |  |
| Fat Free Mass | 28.43 ± 7.59 | 34.33 ± 7.29 | 27.38 ± 7.16 | <0.001 | 26.09 ± 6.65 | 32.28 ± 7.68 | 25.75 ± 6.43 | <0.001 |  |
| Skeletal Muscle Mass | 14.76 ± 4.47 | 18.26 ± 4.28 | 14.15 ± 4.21 | <0.001 | 13.33 ± 3.88 | 16.97 ± 4.46 | 13.13 ± 3.75 | <0.001 |  |
| Percent Body Fat | 24.74 ± 8.54 | 35.11 ± 6.30 | 22.91 ± 7.52 | <0.001 | 23.62 ± 6.86 | 34.20 ± 5.68 | 23.05 ± 6.45 | <0.001 |  |
| Basal Metabolic Rate | 983.97 ± 163.96 | 1111.54 ± 157.48 | 961.47 ± 154.65 | <0.001 | 933.50 ± 143.70 | 1067.07 ± 166.14 | 926.21 ± 138.90 | <0.001 |  |
| Waist-Hip Ratio | 0.77 ± 0.48 | 0.82 ± 0.05 | 0.76 ± 0.03 | <0.001 | 0.77 ± 0.04 | 0.81 ± 0.05 | 0.76 ± 0.03 | <0.001 |  |
| Bone Mineral Content | 1.69 ± 0.48 | 2.06 ± 0.46 | 1.63 ± 0.46 | <0.001 | 1.59 ± 0.44 | 1.98 ± 0.51 | 1.57 ± 0.42 | <0.001 |  |
| Fat Free Mass of Arm | 1.10 ± 0.43 | 1.51 ± 0.44 | 1.03 ± 0.39 | <0.001 | 0.94 ± 0.36 | 1.36 ± 0.44 | 0.92 ± 0.34 | <0.001 |  |
| Fat Free Mass of Trunk | 11.84 ± 3.32 | 14.92 ± 3.22 | 11.30 ± 3.04 | <0.001 | 10.75 ± 2.91 | 13.83 ± 3.25 | 10.58 ± 2.79 | <0.001 |  |
| Fat Free Mass of Leg | 3.97 ± 1.42 | 5.04 ± 1.35 | 3.78 ± 1.34 | <0.001 | 3.49 ± 1.23 | 4.60 ± 1.38 | 3.43 ± 1.20 | <0.001 |  |
| Body Fat Mass of Arm | 0.77 ± 0.48 | 1.44 ± 0.55 | 0.65 ± 0.35 | <0.001 | 0.65 ± 0.33 | 1.27 ± 0.54 | 0.62 ± 0.28 | <0.001 |  |
| Body Fat Mass of Trunk | 4.22 ± 3.57 | 9.16 ± 3.43 | 3.35 ± 2.81 | <0.001 | 3.31 ± 2.62 | 8.07 ± 3.11 | 3.05 ± 2.33 | <0.001 |  |
| Body Fat Mass of Leg | 1.84 ± 0.93 | 3.13 ± 0.89 | 1.61 ± 0.73 | <0.001 | 1.58 ± 0.66 | 2.80 ± 0.83 | 1.51 ± 0.58 | <0.001 |  |
| Percent Body Fat of Arm | 37.86 ± 8.34 | 45.83 ± 5.94 | 36.45 ± 7.90 | <0.001 | 38.95 ± 7.09 | 45.82 ± 5.07 | 38.57 ± 6.99 | <0.001 |  |
| Percent Body Fat of Trunk | 21.47 ± 11.56 | 35.06 ± 8.08 | 19.07 ± 10.37 | <0.001 | 19.78 ± 9.80 | 34.21 ± 7.16 | 18.99 ± 9.30 | <0.001 |  |
| Percent Body Fat of Leg | 29.82 ± 7.30 | 36.65 ± 5.03 | 28.62 ± 6.97 | <0.001 | 29.91 ± 6.51 | 36.60 ± 5.28 | 29.55 ± 6.37 | <0.001 |  |
| Circumference of Neck | 26.66 ± 3.18 | 30.48 ± 2.97 | 25.99 ± 2.71 | <0.001 | 25.40 ± 2.73 | 29.14 ± 2.81 | 25.20 ± 2.57 | <0.001 |  |
| Circumference of Chest | 73.40 ± 8.70 | 83.84 ± 7.94 | 71.56 ± 7.44 | <0.001 | 70.41 ± 7.33 | 80.90 ± 7.57 | 69.83 ± 6.87 | <0.001 |  |
| Circumference of Hip | 82.83 ± 7.72 | 92.29±6.94 | 81.17 ± 6.57 | <0.001 | 79.96 ± 6.15 | 89.56 ± 6.76 | 79.44 ± 5.68 | <0.001 |  |
| Circumference of Arm | 23.38 ± 3.37 | 27.70±2.85 | 22.62 ± 2.84 | <0.001 | 22.08 ± 2.69 | 26.44 ± 2.62 | 21.85 ± 2.48 | <0.001 |  |
| Circumference of Thigh | 44.14 ± 4.97 | 50.16±4.45 | 43.08 ± 4.25 | <0.001 | 42.20 ± 3.83 | 48.27 ± 4.27 | 41.87 ± 3.52 | <0.001 |  |
| Muscle Circumference of Chest | 68.80 ± 7.67 | 77.17 ± 6.94 | 67.32 ± 6.80 | <0.001 | 66.25 ± 6.77 | 74.74 ± 6.73 | 65.79 ± 6.46 | <0.001 |  |
| Muscle Circumference of Abdomen | 58.53 ± 8.15 | 68.44 ± 8.91 | 56.78 ± 6.63 | <0.001 | 56.57 ± 6.67 | 65.81 ± 8.52 | 56.07 ± 6.18 | <0.001 |  |
| Muscle Circumference of Arm | 19.64 ± 2.64 | 22.69 ± 2.20 | 19.11 ± 2.33 | <0.001 | 18.61 ± 2.25 | 21.74 ± 2.02 | 18.44 ± 2.13 | <0.001 |  |
| Muscle Circumference of Thigh | 36.95 ± 4.37 | 41.38 ± 3.84 | 36.17 ± 3.98 | <0.001 | 35.30 ± 3.63 | 39.90 ± 3.83 | 35.04 ± 3.45 | <0.001 |  |
| Fat Thickness of Chest | 0.74 ± 0.25 | 1.07 ± 0.21 | 0.68 ± 0.21 | <0.001 | 0.66 ± 0.19 | 0.99 ± 0.22 | 0.64 ± 0.17 | <0.001 |  |
| Fat Thickness of Abdomen | 0.86 ± 0.29 | 1.24 ± 0.25 | 0.79 ± 0.24 | <0.001 | 0.79 ± 0.22 | 1.15 ± 0.25 | 0.77 ± 0.20 | <0.001 |  |
| Fat Thickness of Arm | 0.60 ± 0.16 | 0.79 ± 0.13 | 0.55 ± 0.13 | <0.001 | 0.55 ± 0.12 | 0.75 ± 0.14 | 0.54 ± 0.11 | <0.001 |  |
| Fat Thickness of Thigh | 1.14 ± 0.22 | 1.40 ± 0.18 | 1.10 ± 0.19 | <0.001 | 1.10 ± 0.17 | 1.34 ± 0.19 | 1.09 ± 0.16 | <0.001 |  |
| Insulin | 6.40 ± 4.03 | 10.53 ± 5.84 | 5.67 ± 3.10 | <0.001 | 6.65 ± 4.64 | 14.58 ± 11.14 | 6.22 ± 3.54 | <0.001 |  |
| Ca | 2.48 ± 0.07 | 2.51 ± 0.07 | 2.48 ± 0.07 | <0.001 | 2.49 ± 0.07 | 2.52 ± 0.10 | 2.49 ± 0.07 | 0.099 |  |
| Phosphate | 1.68 ± 0.13 | 1.68 ± 0.12 | 1.68 ± 0.13 | 0.810 | 1.70 ± 0.13 | 1.68 ± 0.15 | 1.70 ± 0.13 | 0.525 |  |
| Magnesium | 0.85 ± 0.05 | 0.85 ± 0.04 | 0.84 ± 0.05 | 0.053 | 0.84 ± 0.05 | 0.83 ± 0.05 | 0.84 ± 0.05 | 0.509 |  |
| Total bilirubin | 10.11 ± 3.39 | 9.25 ± 2.54 | 10.26 ± 3.50 | 0.023 | 11.02 ± 4.22 | 9.20 ± 2.93 | 11.12 ± 4.26 | 0.013 |  |
| Direct bilirubin | 3.62 ± 1.15 | 3.37 ± 0.94 | 3.67 ± 1.18 | 0.042 | 3.88 ± 1.35 | 3.40 ± 1.00 | 3.91 ± 1.37 | 0.05 |  |
| Total protein | 75.18 ± 3.55 | 76.73 ± 3.39 | 74.91 ± 3.50 | <0.001 | 75.36 ± 3.18 | 75.70 ± 3.42 | 75.34 ± 3.17 | 0.678 |  |
| Albumin | 45.72 ± 1.85 | 46.34 ± 1.50 | 45.61 ± 1.89 | <0.001 | 45.81 ± 1.80 | 45.77 ± 1.29 | 45.82 ± 1.82 | 0.895 |  |
| Alanine aminotransferase | 15.79 ± 9.58 | 21.54 ± 16.55 | 14.78±7.30 | <0.001 | 13.14±4.88 | 14.95 ± 5.35 | 13.04 ± 4.84 | 0.017 |  |
| γ-glutamine transpeptidase | 14.72 ± 4.59 | 18.45 ± 8.13 | 14.06±3.21 | <0.001 | 12.75 ± 2.37 | 13.75 ± 3.75 | 12.69 ± 2.27 | 0.116 |  |
| Asparagine aminotransferase | 26.32 ± 5.91 | 25.34 ± 6.89 | 26.49 ± 5.71 | 0.002 | 25.07 ± 5.36 | 22.18 ± 4.78 | 25.23 ± 5.35 | 0.004 |  |
| Lactate dehydrogenase | 240.83 ± 35.36 | 253.67 ± 35.93 | 238.56 ± 34.81 | <0.001 | 227.63 ± 37.58 | 230.89 ± 39.74 | 227.45 ± 37.49 | 0.325 |  |
| Total bile acid | 3.02 ± 2.10 | 3.00 ± 1.88 | 3.02 ± 2.14 | 0.913 | 2.74 ± 1.96 | 3.30 ± 2.39 | 2.71 ± 1.93 | 0.341 |  |
| Urea | 5.06 ± 0.99 | 5.09 ± 0.96 | 5.06 ± 0.99 | 0.832 | 4.63 ± 0.99 | 4.39 ± 0.86 | 4.64 ± 0.99 | 0.326 |  |
| Creatinine | 45.43 ± 6.31 | 45.85 ± 5.56 | 45.36 ± 6.43 | 0.372 | 44.40 ± 5.82 | 43.57 ± 5.85 | 44.44 ± 5.82 | 0.388 |  |
| Cystatin C | 0.78 ± 0.09 | 0.80 ± 0.08 | 0.78 ± 0.09 | 0.013 | 0.77 ± 0.29 | 0.78 ± 0.09 | 0.76 ± 0.07 | 0.402 |  |
| Retinol-binding protein | 34.23 ± 5.91 | 36.96 ± 5.70 | 33.75 ± 5.82 | <0.001 | 33.60 ± 5.78 | 35.82 ± 6.08 | 33.48 ± 5.74 | 0.050 |  |
| Uric acid | 301.67 ± 71.88 | 341.85 ± 72.46 | 294.58 ± 69.48 | <0.001 | 290.26 ± 54.62 | 338.60 ± 73.69 | 287.62 ± 52.20 | <0.001 |  |
| Globulin | 29.46 ± 2.90 | 30.39 ± 3.09 | 29.30 ± 2.83 | 0.005 | 29.55 ± 2.68 | 29.92 ± 3.26 | 29.53 ± 2.65 | 0.557 |  |
| Asparagine aminotransferase / Alanine aminotransferase | 1.86 ± 0.59 | 1.38 ± 0.48 | 1.94 ± 0.57 | <0.001 | 2.03 ± 0.55 | 1.59 ± 0.49 | 2.06 ± 0.54 | <0.001 |  |
| Uric acid / Globulin | 1.57 ± 0.16 | 1.54 ± 0.16 | 1.57 ± 0.16 | 0.170 | 1.56 ± 0.16 | 1.55 ± 0.18 | 1.56 ± 0.15 | 0.534 |  |
| Urea/ Creatinine | 0.11 ± 0.03 | 0.11 ± 0.02 | 0.11 ± 0.03 | 0.618 | 0.11 ± 0.03 | 0.10 ± 0.03 | 0.11 ± 0.03 | 0.691 |  |
| Total cholesterol | 4.70 ± 0.89 | 4.67 ± 0.86 | 4.71 ± 0.89 | 0.859 | 4.74 ± 0.79 | 4.38 ± 0.65 | 4.76 ± 0.79 | 0.013 |  |
| Lactate dehydrogenase cholesterol | 2.26 ± 0.64 | 2.30 ± 0.64 | 2.26 ± 0.64 | 0.330 | 2.29 ± 0.56 | 2.10 ± 0.56 | 2.30 ± 0.56 | 0.093 |  |
| White blood cells | 6.78 ± 1.69 | 7.39 ± 1.66 | 6.67 ± 1.67 | <0.001 | 6.49 ± 1.51 | 7.21 ± 1.96 | 6.45 ± 1.47 | 0.047 |  |
| Neutrophils% | 46.70 ± 9.34 | 50.70 ± 7.34 | 45.99 ± 9.48 | <0.001 | 47.28 ± 9.77 | 51.23 ± 10.55 | 47.06 ± 9.69 | 0.041 |  |
| Monocyte% | 5.69 ± 1.22 | 5.78 ± 1.16 | 5.68 ± 1.23 | 0.318 | 5.42 ± 1.25 | 5.58 ± 1.12 | 5.41 ± 1.26 | 0.329 |  |
| Lymphocyte% | 43.97 ± 9.18 | 40.30 ± 7.39 | 44.62 ± 9.31 | <0.001 | 44.34 ± 9.68 | 40.50 ± 9.62 | 44.55 ± 9.65 | 0.036 |  |
| Eosinophils% | 3.24 ± 2.03 | 2.85 ± 1.38 | 3.31 ± 2.12 | 0.271 | 2.59 ± 1.81 | 2.36 ± 1.35 | 2.60 ± 1.83 | 0.935 |  |
| Basophil% | 0.40 ± 0.23 | 0.38 ± 0.20 | 0.40 ± 0.24 | 0.768 | 0.37 ± 0.19 | 0.34 ± 0.24 | 0.37 ± 0.19 | 0.080 |  |
| Neutrophils | 3.24 ± 1.35 | 3.80 ± 1.24 | 3.14 ± 1.34 | <0.001 | 3.13 ± 1.30 | 3.84 ± 1.74 | 3.09 ± 1.26 | 0.027 |  |
| Monocyte | 0.38 ± 0.12 | 0.43 ± 0.13 | 0.38 ± 0.12 | <0.001 | 0.35 ± 0.10 | 0.40 ± 0.11 | 0.35 ± 0.10 | 0.009 |  |
| Lymphocytes | 2.91 ± 0.72 | 2.92 ± 0.63 | 2.91 ± 0.74 | 0.788 | 2.82 ± 0.69 | 2.79 ± 0.59 | 2.82 ± 0.69 | 0.887 |  |
| Eosinophils | 0.22 ± 0.15 | 0.21 ± 0.12 | 0.22 ± 0.15 | 0.724 | 0.17 ± 0.13 | 0.16 ± 0.09 | 0.17 ± 0.13 | 0.602 |  |
| Basophil | 0.03 ± 0.02 | 0.03 ± 0.02 | 0.03 ± 0.02 | 0.246 | 0.02 ± 0.01 | 0.02 ± 0.02 | 0.02 ± 0.01 | 0.436 |  |
| Red blood cell count | 4.84 ± 0.32 | 4.89 ± 0.35 | 4.84 ± 0.31 | 0.222 | 4.76 ± 0.32 | 4.78 ± 0.31 | 4.76 ± 0.32 | 0.769 |  |
| Hemoglobin | 135.38 ± 7.00 | 135.80 ± 7.75 | 135.30 ± 6.86 | 0.739 | 134.61 ± 6.86 | 135.18 ± 8.21 | 134.58 ± 6.79 | 0.466 |  |
| Hematocrit | 41.08 ± 2.26 | 41.35 ± 2.47 | 41.04 ± 2.22 | 0.394 | 41.14 ± 2.22 | 41.40 ± 2.41 | 41.12 ± 2.21 | 0.49 |  |
| Mean corpuscular volume | 84.94 ± 3.49 | 84.74 ± 3.36 | 84.98 ± 3.51 | 0.614 | 86.51 ± 3.87 | 86.75 ± 2.52 | 86.50 ± 3.93 | 0.844 |  |
| Mean corpuscular hemoglobin | 28.00 ± 1.37 | 27.85 ± 1.39 | 28.03 ± 1.36 | 0.168 | 28.33 ± 1.52 | 28.34 ± 1.09 | 28.33 ± 1.54 | 0.750 |  |
| Mean corpuscular hemoglobin concentration | 329.69 ± 8.16 | 328.59 ± 8.48 | 329.88 ± 8.10 | 0.180 | 327.37 ± 8.09 | 326.57 ± 9.47 | 327.42 ± 8.02 | 0.586 |  |
| Red blood cell distribution width | 13.11 ± 0.59 | 13.25 ± 0.54 | 13.09 ± 0.60 | 0.004 | 12.84 ± 0.53 | 12.82 ± 0.46 | 12.84 ± 0.53 | 0.923 |  |
| Platelets | 308.42 ± 63.08 | 323.74 ± 65.93 | 305.72 ± 62.25 | 0.013 | 301.84 ± 61.00 | 337.64 ± 67.08 | 299.89 ± 60.11 | 0.003 |  |
| Plateletocrit | 0.31 ± 0.06 | 0.33 ± 0.06 | 0.31 ± 0.05 | <0.001 | 0.30 ± 0.06 | 0.33 ± 0.06 | 0.30 ± 0.05 | <0.001 |  |
| Mean platelet volume | 10.09 ± 1.07 | 10.16 ± 0.94 | 10.07 ± 1.09 | 0.233 | 10.02 ± 0.99 | 9.95 ± 1.11 | 10.02 ± 0.99 | 0.682 |  |
| Platelet distribution width | 15.92 ± 0.03 | 15.93 ± 0.28 | 15.92 ± 0.31 | 0.422 | 15.92 ± 0.03 | 15.90 ± 0.29 | 15.92 ± 0.30 | 0.852 |  |
| Urine occult blood |  |  |  | 0.002 |  |  |  | 0.712 |  |
| - | 540(95.24%) | 75(88.24%) | 465(96.47%) |  | 490(90.57%) | 25(89.29%) | 465(90.64%) |  |  |
| ± | 23(4.06%) | 7(8.24%) | 52(5.23%) |  | 39(7.21%) | 3(10.71%) | 36(7.02%) |  |  |
| 1+ | 4(0.71%) | 3(353%) | 10(1.01%) |  | 9(1.66%) | 0(0.00%) | 9(1.75%) |  |  |
| 3+ | 0(0.00%) | 0(0.00%) | 3(0.30%) |  | 3(0.55%) | 0(0.00%) | 3(0.58%) |  |  |
| Urobilinogen |  |  |  | 0.512 |  |  |  | 0.554 |  |
| - | 553(97.53%) | 82(96.47%) | 471(97.72%) |  | 526(97.23%) | 27(96.43%) | 499(97.27%) |  |  |
| 1+ | 13(2.29%) | 3(3.53%) | 10(2.07%) |  | 13(2.40%) | 1(3.57%) | 12(2.34$) |  |  |
| 2+ | 1(0.18%) | 0(0.00%) | 1(0.21%) |  | 2(0.37%) | 0(0.00%) | 2(0.39%) |  |  |
| Protein |  |  |  | 1 |  |  |  | 0.334 |  |
| - | 561(99.12%) | 85(100.00%) | 476(98.96%) |  | 520(96.47%) | 26(92.86%) | 494(96.67%) |  |  |
| ± | 3(0.53%) | 0(0.00%) | 3(0.62%) |  | 10(1.86%) | 1(3.57%) | 9(1.76%) |  |  |
| 1+ | 2(0.35%) | 0(0.00%) | 2(0.42%) |  | 9(1.67%) | 1(3.57%) | 8(1.57%) |  |  |
| Vitamin C |  |  |  | 0.588 |  |  |  | 0.208 |  |
| - | 469(82.72%) | 75(88.24%) | 394(81.74%) |  | 458(84.66%) | 23(82.14%) | 435(84.80%) |  |  |
| ± | 29(5.11%) | 2(2.35%) | 27(5.60%) |  | 30(5.55%) | 0(0.00%) | 30(5.85%) |  |  |
| 1+ | 24(4.23%) | 2(2.35%) | 22(4.56%) |  | 17(3.14%) | 2(7.14%) | 15(2.92%) |  |  |
| 2+ | 15(2.65%) | 1(1.18%) | 14(2.09%) |  | 16(2.96%) | 1(3.57%) | 15(2.92%) |  |  |
| 3+ | 30(5.29%) | 5(5.88%) | 25(5.19%) |  | 20(3.70%) | 2(7.14%) | 18(3.51%) |  |  |
| Leukocyte esterase |  |  |  | 0.726 |  |  |  | 0.423 |  |
| - | 532(93.38%) | 79(92.94%) | 453(93.98%) |  | 258(47.69%) | 17(60.71%) | 241(46.98%) |  |  |
| ± | 29(5.11%) | 5(5.88%) | 24(4..98%) |  | 119(22.00%) | 3(10.71%) | 116(22.61%) |  |  |
| 1+ | 4(0.71%) | 1(1.18%) | 3(0.62%) |  | 86(15.90%) | 3(10.71%) | 83(16.18%) |  |  |
| 2+ | 2(0.35%) | 0(0.00%) | 2(0.41%) |  | 48(8.87%) | 3(10.71%) | 45(8.77%) |  |  |
| 3+ | 0(0.00%) | 0(0.00%) | 0(0.00%) |  | 30(5.55%) | 2(7.14%) | 28(5.46%) |  |  |
| Urinary calcium |  |  |  | 0.016 |  |  |  | 0.287 |  |
| <1.00 | 84(14.81%) | 21(24.71%) | 63(13.07%) |  | 55(10.17%) | 5(17.86%) | 50(9.75%) | 0.919 |  |
| 2.5 | 386(68.08%) | 48(56.47%) | 338(70.12%) |  | 384(70.98%) | 17(60.71%) | 367(71.54%) | 0.306 |  |
| 5 | 97(17.11%) | 16(18.82%) | 81(16.80%) |  | 102(18.85%) | 6(21.43%) | 96(18.71%) |  |  |
| Urine red blood cells | 2.00 ± 3.76 | 2.67 ± 5.20 | 1.88 ± 3.44 | 0.246 | 3.24 ± 5.54 | 3.57 ± 5.51 | 3.22 ± 5.55 |  |  |
| Urine white blood cells | 2.65 ± 4.41 | 3.02 ± 3.57 | 2.59 ± 4.55 | 0.034 | 15.30 ± 31.32 | 20.61 ± 36.24 | 15.01 ± 31.05 | <0.001 |  |
| Urine protein | 0.09 ± 0.07 | 0.10 ± 0.05 | 0.09 ± 0.07 | 0.007 | 0.09 ± 0.07 | 0.09 ± 0.08 | 0.09 ± 0.07 | 0.93 |  |
| Urinary protein creatinine ratio | 0.07 ± 0.02 | 0.06 ± 0.02 | 0.07 ± 0.02 | 0.157 | 0.08 ± 0.04 | 0.07 ± 0.04 | 0.08 ± 0.04 | 0.258 |  |
| Urine creatinine | 10874.96 ± 4628.96 | 12557.94 ± 4550.91 | 10578.17 ± 4593.46 | <0.001 | 10229.25 ± 4713.25 | 10322.75 ± 3768.06 | 10224.15 ± 4762.39 | 0.63 |  |
| Urine specific gravity | 1.02 ± 0.01 | 1.02 ± 0.01 | 1.02 ± 0.01 | <0.001 | 1.02 ± 0.01 | 1.02 ± 0.01 | 1.02 ± 0.01 | 0.206 |  |
| Potential of hydrogen | 6.04 ± 0.54 | 5.89 ± 0.51 | 6.07 ± 0.54 | 0.003 | 6.11 ± 0.53 | 5.79 ± 0.52 | 6.13 ± 0.53 | <0.001 |  |
| Epithelial cell count | 0.98 ± 2.06 | 1.00 ± 1.70 | 0.98 ± 2.12 | 0.357 | 6.22 ± 14.96 | 11.56 ± 26.97 | 5.92 ± 14.00 | 0.439 |  |
| Urine mucous strands | 27.38 ± 55.07 | 27.75 ± 35.72 | 27.31 ± 57.84 | 0.197 | 24.30 ± 48.59 | 22.91 ± 31.42 | 24.38 ± 49.38 | 0.819 |  |

Data are presented as N (%) or the mean ± standard deviation. * denotes a total of 674 participants, with 94 in the MAFLD group and 580 in the non-MAFLD group.

MAFLD, metabolic dysfunction-associated fatty liver disease; %, percentage; /, ratio.

**Table S4. Correlation coefficient between MAFLD diagnostic biomarkers and less-explored biomarkers.**

| **Diagnosis variables** | **Other variables** | ***r*** | ***p-value*** |
| --- | --- | --- | --- |
| BMI | Body Fat Mass | 0.943 | <0.001 |
|  | Percent Body Fat | 0.830 | <0.001 |
|  | Body Fat Mass of Arm | 0.902 | <0.001 |
|  | Body Fat Mass of Trunk | 0.944 | <0.001 |
|  | Percent Body Fat of Trunk | 0.868 | <0.001 |
|  | Body Fat Mass of Leg | 0.941 | <0.001 |
|  | Circumference of Neck | 0.896 | <0.001 |
|  | Circumference of Chest | 0.907 | <0.001 |
|  | Circumference of Abdomen | 0.883 | <0.001 |
|  | Circumference of Hip | 0.927 | <0.001 |
|  | Circumference of Arm | 0.975 | <0.001 |
|  | Circumference of Thigh | 0.926 | <0.001 |
|  | Muscle Circumference of Chest | 0.842 | <0.001 |
|  | Muscle Circumference of Abdomen | 0.812 | <0.001 |
|  | Muscle Circumference of Arm | 0.917 | <0.001 |
|  | Muscle Circumference of Thigh | 0.818 | <0.001 |
|  | Fat Thickness of Chest | 0.882 | <0.001 |
|  | Fat Thickness of Abdomen | 0.849 | <0.001 |
|  | Fat Thickness of Arm | 0.838 | <0.001 |
| Circumference of Abdomen | Body Fat Mass | 0.940 | <0.001 |
|  | Soft Lean Mass | 0.851 | <0.001 |
|  | Fat Free Mass | 0.852 | <0.001 |
|  | Skeletal Muscle Mass | 0.853 | <0.001 |
|  | Fat Free Mass of Arm | 0.901 | <0.001 |
|  | Fat Free Mass of Trunk | 0.904 | <0.001 |
|  | Fat Free Mass of Leg | 0.829 | <0.001 |
|  | Body Fat Mass of Arm | 0.930 | <0.001 |
|  | Body Fat Mass of Trunk | 0.949 | <0.001 |
|  | Body Fat Mass of Leg | 0.911 | <0.001 |
|  | Basal Metabolic Rate | 0.852 | <0.001 |
|  | Waist-Hip Ratio | 0.893 | <0.001 |
|  | Bone Mineral Content | 0.844 | <0.001 |
|  | Circumference of Neck | 0.924 | <0.001 |
|  | Circumference of Chest | 0.966 | <0.001 |
|  | Circumference of Hip | 0.957 | <0.001 |
|  | Circumference of Arm | 0.926 | <0.001 |
|  | Circumference of Thigh | 0.937 | <0.001 |
|  | Muscle Circumference of Chest | 0.947 | <0.001 |
|  | Muscle Circumference of Abdomen | 0.986 | <0.001 |
|  | Muscle Circumference of Arm | 0.928 | <0.001 |
|  | Muscle Circumference of Thigh | 0.905 | <0.001 |

**Table S5. Correlation coefficient between MAFLD diagnostic biomarkers and routine blood biomarkers.**

|  | WBC | NE% | MO% | LY% | EO% | BA% | NE | MO | LY | EO | BA | RBC | HGB | HCT | MCV | MCH | MCHC | RDW | PLT | PCT | MPV |
| --- | --- | --- | --- | --- | --- | --- | --- | --- | --- | --- | --- | --- | --- | --- | --- | --- | --- | --- | --- | --- | --- |
| NE% | 0.43* |  |  |  |  |  |  |  |  |  |  |  |  |  |  |  |  |  |  |  |  |
| MO% | -0.13* | -0.02 |  |  |  |  |  |  |  |  |  |  |  |  |  |  |  |  |  |  |  |
| LY% | -0.42* | -0.97* | -0.11* |  |  |  |  |  |  |  |  |  |  |  |  |  |  |  |  |  |  |
| EO% | 0.02 | -0.18* | -0.01 | -0.03 |  |  |  |  |  |  |  |  |  |  |  |  |  |  |  |  |  |
| BA% | -0.15* | -0.20* | 0.06* | 0.12* | 0.26* |  |  |  |  |  |  |  |  |  |  |  |  |  |  |  |  |
| NE | 0.87* | 0.79* | -0.08* | -0.76* | -0.10* | -0.21* |  |  |  |  |  |  |  |  |  |  |  |  |  |  |  |
| MO | 0.70* | 0.33* | 0.59* | -0.41* | 0.01 | -0.07* | 0.63* |  |  |  |  |  |  |  |  |  |  |  |  |  |  |
| LY | 0.46* | -0.53* | -0.23* | 0.56* | 0.03 | 0.00 | -0.02 | 0.20* |  |  |  |  |  |  |  |  |  |  |  |  |  |
| EO | 0.30* | -0.06* | -0.05 | -0.13* | 0.94* | 0.21* | 0.14* | 0.20* | 0.19* |  |  |  |  |  |  |  |  |  |  |  |  |
| BA | 0.20* | -0.07* | 0.02 | -0.01 | 0.29* | 0.89* | 0.07* | 0.17* | 0.21* | 0.35* |  |  |  |  |  |  |  |  |  |  |  |
| RBC | 0.16* | 0.05 | -0.02 | -0.05 | 0.00 | 0.01 | 0.12* | 0.10* | 0.11* | 0.06 | 0.08* |  |  |  |  |  |  |  |  |  |  |
| HGB | 0.13* | 0.08* | -0.04 | -0.07* | 0.00 | -0.04 | 0.12* | 0.07* | 0.06 | 0.04 | 0.01 | 0.58* |  |  |  |  |  |  |  |  |  |
| HCT | 0.15* | 0.08* | -0.02 | -0.08* | -0.01 | -0.01 | 0.13* | 0.09* | 0.07* | 0.04 | 0.05 | 0.71* | 0.89* |  |  |  |  |  |  |  |  |
| MCV | -0.06* | 0.02 | 0.00 | -0.01 | -0.02 | -0.02 | -0.03 | -0.05 | -0.07* | -0.03 | -0.05 | -0.58* | 0.20* | 0.16* |  |  |  |  |  |  |  |
| MCH | -0.08* | 0.00 | -0.01 | 0.00 | -0.01 | -0.05 | -0.05 | -0.07* | -0.08* | -0.03 | -0.08* | -0.66* | 0.22* | -0.04 | 0.88* |  |  |  |  |  |  |
| MCHC | -0.05 | -0.02 | -0.03 | 0.03 | 0.02 | -0.06 | -0.04 | -0.06* | -0.04 | 0.00 | -0.09* | -0.35* | 0.12* | -0.34* | 0.08* | 0.55* |  |  |  |  |  |
| RDW | 0.08* | 0.09* | 0.00 | -0.09* | 0.02 | 0.00 | 0.08* | 0.07* | 0.01 | 0.04 | 0.03 | 0.31* | -0.17* | -0.06 | -0.48* | -0.51* | -0.24* |  |  |  |  |
| PLT | 0.25* | 0.02 | 0.02 | -0.03 | 0.01 | 0.14* | 0.16* | 0.22* | 0.23* | 0.08* | 0.23* | 0.02 | -0.07* | -0.04 | -0.08* | -0.10* | -0.05 | -0.04 |  |  |  |
| PCT | 0.27* | 0.04 | 0.03 | -0.05 | -0.01 | 0.14* | 0.18* | 0.24* | 0.24* | 0.07* | 0.24* | 0.07* | -0.06 | 0.02 | -0.08* | -0.14* | -0.15* | 0.02 | 0.87* |  |  |
| MPV | -0.02 | 0.03 | 0.01 | -0.02 | -0.04 | -0.02 | 0.00 | -0.02 | -0.04 | -0.04 | -0.04 | 0.07* | 0.02 | 0.10* | 0.02 | -0.07* | -0.18* | 0.12* | -0.44* | 0.05 |  |
| PDW | -0.07* | 0.05 | 0.03 | -0.04 | -0.06* | -0.10* | -0.01 | -0.03 | -0.11* | -0.07* | -0.13* | 0.02 | 0.15* | 0.14* | 0.12* | 0.10* | 0.01 | -0.01 | -0.42* | -0.11* | 0.65* |

WBC, white blood cells; NE, neutrophils; MO, monocyte; LY, lymphocytes; EO, eosinophils; BA, basophil; RBC, red blood cell count; HGB, hemoglobin; HCT, hematocrit; MCV, mean corpuscular volume; MCH, mean corpuscular hemoglobin; MCHC, mean corpuscular hemoglobin concentration; RDW, red blood cell distribution width; PLT, platelets; PCT, platelet count; MPV, mean platelet volume; PDW, platelet distribution width; %, percentage; * denotes *p-value* < 0.05.

**Table S6. Correlation coefficient between MAFLD diagnostic biomarkers and blood biochemical biomarkers.**

|  | INS | CA | PHOS | MG | TBIL | DBIL | TP | ALB | ALT | GGT | AST | LDH | TBA | UREA | CREA | CYSC | RBP | UA | GLU | TG | TCHOL | LDLC | HDLC | G | AST/ALT | A/G |
| --- | --- | --- | --- | --- | --- | --- | --- | --- | --- | --- | --- | --- | --- | --- | --- | --- | --- | --- | --- | --- | --- | --- | --- | --- | --- | --- |
| CA | 0.05 |  |  |  |  |  |  |  |  |  |  |  |  |  |  |  |  |  |  |  |  |  |  |  |  |  |
| PHOS | -0.03 | 0.06* |  |  |  |  |  |  |  |  |  |  |  |  |  |  |  |  |  |  |  |  |  |  |  |  |
| MG | 0.03 | 0.09* | 0.07* |  |  |  |  |  |  |  |  |  |  |  |  |  |  |  |  |  |  |  |  |  |  |  |
| TBIL | -0.13* | 0.02 | 0.00 | 0.03 |  |  |  |  |  |  |  |  |  |  |  |  |  |  |  |  |  |  |  |  |  |  |
| DBIL | -0.09* | -0.01 | -0.01 | 0.01 | 0.96* |  |  |  |  |  |  |  |  |  |  |  |  |  |  |  |  |  |  |  |  |  |
| TP | 0.13* | 0.38* | -0.11* | 0.14* | 0.01 | -0.03 |  |  |  |  |  |  |  |  |  |  |  |  |  |  |  |  |  |  |  |  |
| ALB | 0.07* | 0.55* | -0.04 | 0.18* | 0.12* | 0.09* | 0.56* |  |  |  |  |  |  |  |  |  |  |  |  |  |  |  |  |  |  |  |
| ALT | 0.16* | 0.03 | -0.03 | 0.07* | -0.06* | -0.06* | 0.12* | 0.03 |  |  |  |  |  |  |  |  |  |  |  |  |  |  |  |  |  |  |
| GGT | 0.32* | 0.12* | 0.00 | 0.15* | -0.05 | -0.05 | 0.22* | 0.14* | 0.55* |  |  |  |  |  |  |  |  |  |  |  |  |  |  |  |  |  |
| AST | -0.28* | 0.06 | 0.00 | 0.02 | -0.01 | -0.06* | 0.06* | -0.03 | 0.55* | 0.19* |  |  |  |  |  |  |  |  |  |  |  |  |  |  |  |  |
| LDH | -0.04 | 0.02 | 0.03 | 0.13* | 0.00 | -0.06 | 0.11* | 0.04 | 0.22* | 0.18* | 0.39* |  |  |  |  |  |  |  |  |  |  |  |  |  |  |  |
| TBA | 0.02 | 0.04 | 0.00 | 0.00 | -0.11* | -0.09* | -0.07* | 0.00 | 0.02 | -0.03 | 0.05 | 0.03 |  |  |  |  |  |  |  |  |  |  |  |  |  |  |
| UREA | -0.13* | 0.03 | 0.03 | 0.05 | -0.07* | -0.11* | 0.08* | 0.10* | 0.06* | 0.04 | 0.13* | 0.07* | 0.12* |  |  |  |  |  |  |  |  |  |  |  |  |  |
| CREA | 0.17* | -0.14* | -0.08* | 0.17* | 0.09* | 0.12* | 0.03 | 0.07* | -0.04 | 0.11* | -0.17* | 0.02 | -0.01 | 0.08* |  |  |  |  |  |  |  |  |  |  |  |  |
| CYSC | 0.15* | 0.01 | 0.19* | 0.10* | 0.12* | 0.14* | -0.05 | -0.07* | 0.05 | 0.15* | -0.02 | 0.09* | -0.02 | 0.04 | 0.50* |  |  |  |  |  |  |  |  |  |  |  |
| RBP | 0.26* | 0.17* | 0.07* | 0.15* | 0.03 | 0.01 | 0.10* | 0.22* | 0.09* | 0.30* | -0.12* | 0.01 | -0.04 | 0.12* | 0.26* | 0.26* |  |  |  |  |  |  |  |  |  |  |
| UA | 0.28* | 0.07* | 0.05 | 0.07* | 0.06* | 0.10* | 0.13* | 0.11* | 0.13* | 0.32* | -0.12* | 0.11* | -0.06* | -0.01 | 0.30* | 0.34* | 0.36* |  |  |  |  |  |  |  |  |  |
| GLU | 0.41* | 0.09* | -0.04 | 0.07* | -0.06* | -0.05 | 0.11* | 0.12* | 0.06 | 0.15* | -0.18* | 0.04 | 0.01 | -0.04 | 0.15* | 0.08* | 0.16* | 0.08* |  |  |  |  |  |  |  |  |
| TG | 0.38* | 0.16* | 0.00 | 0.11* | -0.09* | -0.12* | 0.13* | 0.07* | 0.11* | 0.24* | -0.07* | 0.02 | 0.03 | -0.14* | 0.04 | 0.17* | 0.29* | 0.18* | 0.12* |  |  |  |  |  |  |  |
| TCHOL | -0.13* | 0.19* | 0.00 | 0.08* | 0.09* | -0.11* | 0.18* | 0.15* | 0.02 | 0.08* | 0.16* | 0.09* | -0.04 | 0.15* | -0.09* | -0.04 | 0.12* | -0.08* | -0.05 | 0.07* |  |  |  |  |  |  |
| LDLC | -0.12* | 0.16* | 0.02 | 0.06 * | 0.04 | -0.14* | 0.13* | 0.07* | 0.04 | 0.10* | 0.15* | 0.07* | -0.03 | 0.14* | -0.14* | -0.05 | 0.10* | -0.06 | -0.08* | 0.07* | 0.91* |  |  |  |  |  |
| HDLC | -0.34* | 0.08* | 0.05 | -0.05 | 0.16* | 0.06* | -0.04 | 0.07* | -0.13* | -0.18* | 0.18* | -0.01 | 0.01 | 0.14* | -0.11* | -0.10* | -0.09* | -0.27* | -0.15* | -0.39* | 0.45* | 0.13* |  |  |  |  |
| G | 0.11* | 0.10* | -0.10* | 0.05 | -0.06* | -0.09* | 0.84* | 0.02 | 0.12* | 0.17* | 0.09* | 0.10* | -0.09* | 0.03 | 0.00 | -0.01 | -0.02 | 0.09* | 0.06 | 0.12* | 0.11* | 0.11* | -0.09* |  |  |  |
| AST/ALT | -0.35* | 0.01 | 0.02 | -0.08* | 0.06 | 0.03 | -0.08* | -0.05 | -0.55* | -0.34* | 0.16* | -0.05 | 0.02 | -0.05 | -0.08* | -0.08* | -0.24* | -0.25* | -0.18* | -0.17* | 0.05 | 0.02 | 0.25* | -0.07* |  |  |
| A/G | -0.07 | 0.12* | 0.08* | 0.02 | 0.10* | 0.13* | -0.56* | 0.36* | -0.10* | -0.10* | -0.10* | -0.08* | 0.09* | 0.01 | 0.04 | 0.00 | 0.10* | -0.03 | -0.01 | -0.08* | -0.05 | -0.08* | 0.11* | -0.91* | 0.04 |  |
| UREA/CREA | -0.20* | 0.11* | 0.08* | -0.04 | -0.12* | -0.16* | 0.04 | 0.05 | 0.08* | -0.02 | 0.20* | 0.04 | 0.11* | 0.82* | -0.48* | -0.24* | -0.03 | -0.17* | -0.12* | -0.14* | 0.18* | 0.19* | 0.18* | 0.01 | 0.00 | 0.00 |

INS, insulin; Ca, calcium; PHOS, phosphate; Mg, magnesium; TBIL, total bilirubin; DBIL, directbilirubin; TP, total protein; ALB, albumin; ALT, alanine aminotransferase; GGT, γ-glutamine transpeptidase; AST, asparagine aminotransferase; LDH, lactate dehydrogenase; TBA, total bile acid; Cr, creatinine; CysC, cystatin C; RBP, retinol-binding protein; UA, uric acid; G, globulin; TCHOL, total cholesterol; LDHC, lactate dehydrogenase cholesterol; /, ratio; * denotes *p-value* < 0.05.

**Table S7. Correlation coefficient between MAFLD diagnostic biomarkers and routine urine biomarkers.**

|  | URBC | UWBC | UPRO | UPCR | UCREA | SG | PH | EC |
| --- | --- | --- | --- | --- | --- | --- | --- | --- |
| UWBC | 0.40* |  |  |  |  |  |  |  |
| UPRO | 0.26* | 0.13* |  |  |  |  |  |  |
| UPCR | 0.19* | 0.13* | 0.74* |  |  |  |  |  |
| UCREA | 0.19* | 0.09* | 0.72* | 0.19* |  |  |  |  |
| USG | 0.12* | 0.08* | 0.47* | 0.13* | 0.69* |  |  |  |
| PH | 0.00 | -0.05 | -0.08* | 0.07* | -0.21* | -0.54* |  |  |
| EC | 0.22* | 0.27* | 0.16* | 0.15* | 0.10* | 0.03 | -0.03 |  |
| MUCUS | 0.09* | 0.06 | 0.52* | 0.23* | 0.55* | 0.42* | -0.18* | 0.06 |

URBC, urine red blood cells; UWBC, urine white blood cells; UPRO, urine protein; UPCR, urinary protein creatinine ratio; UCREA, urine creatinine; USG, urine specific gravity; pH, potential of hydrogen; EC, epithelial cell count; * denotes *p-value* < 0.05.
